# A nonsense mutation of human XRCC4 is associated with adult-onset progressive encephalocardiomyopathy

**DOI:** 10.15252/emmm.201404803

**Published:** 2015-04-14

**Authors:** Leonardo Bee, Alessia Nasca, Alice Zanolini, Filippo Cendron, Pio d'Adamo, Rodolfo Costa, Costanza Lamperti, Lucia Celotti, Daniele Ghezzi, Massimo Zeviani

**Affiliations:** 1 Department of Biology, University of PaduaPadua, Italy; 2 Molecular Neurogenetics Unit, Foundation IRCCS Institute of Neurology “Carlo Besta”Milan, Italy; 3 Department of Medical Sciences, University of TriesteTrieste, Italy; 4 MRC Mitochondrial Biology Unit, CB2 0XYCambridge, UK

**Keywords:** DNA repair, encephalocardiomyopathy, NHEJ, XRCC4

## Abstract

We studied two monozygotic twins, born to first cousins, affected by a multisystem disease. At birth, they both presented with bilateral cryptorchidism and malformations. Since early adulthood, they developed a slowly progressive neurological syndrome, with cerebellar and pyramidal signs, cognitive impairment, and depression. Dilating cardiomyopathy is also present in both. By whole-exome sequencing, we found a homozygous nucleotide change in *XRCC4* (c.673C>T), predicted to introduce a premature stop codon (p.R225*). *XRCC4* transcript levels were profoundly reduced, and the protein was undetectable in patients' skin fibroblasts. XRCC4 plays an important role in non-homologous end joining of DNA double-strand breaks (DSB), a system that is involved in repairing DNA damage from, for example, ionizing radiations. Gamma-irradiated mutant cells demonstrated reduction, but not abolition, of DSB repair. In contrast with embryonic lethality of the *Xrcc4* KO mouse, nonsense mutations in human XRCC4 have recently been associated with primordial dwarfism and, in our cases, with adult-onset neurological impairment, suggesting an important role for DNA repair in the brain. Surprisingly, neither immunodeficiency nor predisposition to malignancy was reported in these patients.

See also: **JP de Villartay** (July 2015)

## Introduction

DNA breaks are severe injuries for cells, potentially leading to cell cycle arrest and eventually death, accelerated aging, or oncogenic transformation. During evolution, different DNA repair pathways have evolved in order to minimize the deleterious impact of these lesions, affecting either single or double-strand DNA (ssDNA, dsDNA). These mechanisms include base excision repair (BER), nucleotide excision repair (NER), and double-strand break repair (DSBR) systems.

Two main DSBR pathways are known: homologous recombination (HR) (San Filippo *et al*, 2008) and non-homologous end joining (NHEJ) (Lieber, 2010). While HR results in an error-free repair, NHEJ is an intrinsically error-prone pathway. HR is a process predominantly exploited by lower organisms, whereas both HR and NHEJ concur to maintain chromosomal integrity in eukaryotes. In mammals, particularly in human cells, the majority of DSBs are repaired by NHEJ. The preferential involvement and efficiency of NHEJ and HR repair systems vary during the different phases of the cell cycle: NHEJ is active in all the cell cycle phases, whereas HR is active only during the S and G2 phases. In fact, since HR relies on the availability of undamaged templates to restore any lost sequence information, it can operate only if a sister chromatid in close contact to the damaged one is ready to be used as a template. Hence, in cell cycle phases other than S/G2, since no sister chromatid is available, NHEJ is the only option for cells to repair DSBs.

Non-homologous end joining is carried out by two main complexes (Lieber, 2010): the DNA-dependent protein kinase (DNA-PK) holoenzyme, formed by the KU70/KU80 heterodimer bound to the DNA-PK catalytic subunit, and the DNA LIG4-XLF-XRCC4 complex; the former complex has a major regulatory role (Meek *et al*, 2007), whereas the latter possesses the catalytic activity responsible for DNA rejoining. DNA ligase IV (LIG4) is the NHEJ-specific ligase; its stability is increased by interaction with the X-ray repair cross complementing-4 (XRCC4) protein (Bryans *et al*, 1999). A further component of the ligation complex is XLF/Cernunnos, although it has been reported to be dispensable for DNA LIG4 stability (Riballo *et al*, 2009). Very recently an additional protein, PAXX (PAralog of XRCC4 and XLF, also called C9orf142), has been described as a component of this machinery, with a role in binding KU70/KU80 and promoting DSBR through stabilization of NHEJ protein assembly at DSB sites (Ochi *et al*, 2015). An alternative NHEJ (A-NHEJ) process has been identified that does not require the same proteins/enzymes used by Ku-dependent C-NHEJ (classic non-homologous end joining) and that contributes to DNA rejoining in the absence of DNA ligase IV (Wang *et al*, 2003; Goodarzi & Jeggo, 2013).

The most severe DNA damage produced by ionizing radiation (IR) consists in double-strand breaks.

Non-homologous end joining represents the major mechanism for repairing two-ended DSBs such as those generated by IR, but HR also plays a role, particularly in cycling cells during S/G2 phases.

Using whole-exome sequencing, we identified a *XRCC4* nonsense mutation, in two identical twin brothers born to consanguineous parents.

### Case reports

**Figure 1:**
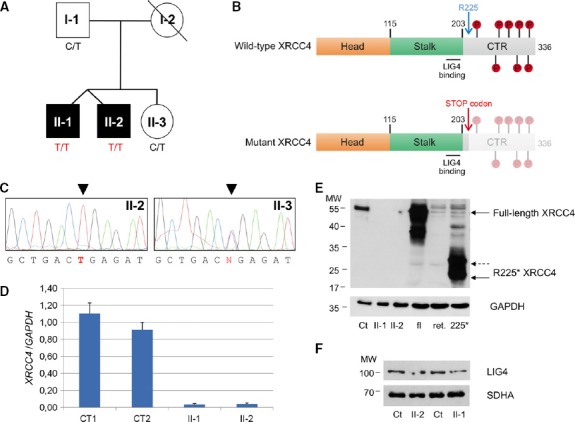
Genetic and molecular features of *XRCC4*-mutant patients

We studied two 50-year-old twin brothers (II-1 and II-2), born to first-degree cousins. Their father, now in his seventies, is alive and well; their mother committed suicide at 55 years. No information is available on her psychiatric status before the event. A 47-year-old sister (II-3) is alive and well (pedigree in Fig1A).

Pedigree. Black symbols designate affected subjects.

Schematic view of wild-type and mutant (R225*) XRCC4 proteins with their main domains and phosphorylation sites. The numbering refers to amino acids of refseq: NP_071801.1. CTR: C-terminal region.

Electropherograms of the *XRCC4* genomic region encompassing the nucleotide substitutions in available members of the family.

Quantitative real-time PCR of *XRCC4* relative to *GAPDH* mRNA in affected (II-1 and II-2) and control (CT1 and CT2) fibroblasts. Each value refers to the mean of three independent experiments performed in duplicate. Error bars indicate the standard deviation.

Immunoblot analysis of total lysates from fibroblasts (fbs) and of *in vitro*-synthesized proteins (i.v.p). fl: *in vitro*-translated full-length XRCC4 protein; ret.: reticulocyte lysate used for *in vitro* protein synthesis; 225*: *in vitro*-translated XRCC4 R225* truncated protein; Ct: lysate of control fbs; and II-1 and II-2: lysates of fbs from patient II-1 and II-2. α-XRCC4 (Abcam, 1:1,000) and α-GAPDH antibodies were used.

Immunoblot analysis of total lysates from fibroblasts using α-LIG4 (GeneTex, 1:1,000) and α-SDHA antibodies. Ct: lysate of control fbs; II-1 and II-2: lysates of fbs from patient II-1 and II-2. The position of the molecular weight (MW) marker proteins is indicated.

#### II-1

Since birth, he presented with bilateral cryptorchidism, hypotelorism, short limbs, pes cavus, and short stature. Alcohol addiction is reported in young adulthood. At 27 years of age, he was diagnosed a dilated cardiomyopathy, with hypertrophy of interventricular septum and a residual ejection fraction of 25%; this condition was attributed to his relevant alcohol intake, and treated with anti-arrhythmic, anti-aggregant, and anti-hypertensive drugs. Since 44 years of age, he carries an ICD (implantable cardioverter defibrillator).

He reported gait difficulties and motor imbalance since 30 years of age; at 46 years he experienced sudden worsening of gait described as an “impaired control” of leg movements, which became progressive over time.

At the time of our neurological assessment, subject II-1 (aged 48) showed cognitive impairment (inability to count backwards), nystagmus, and slowing of eye pursuits, dysarthria, dysmetria, positive sensitized Romberg maneuver, and wide-based and camptocormic gait with slight steppage and diffuse pyramidal signs, requiring a cane for deambulation.

Routine blood laboratory tests did not show alterations besides glucose intolerance (fasting blood glucose 120 mg/dl, normal values, n.v. 50–110), moderately elevated total cholesterol (238 mg/dl, n.v. 110–200), a slight increase in alpha-fetoprotein (5.65 ng/ml, n.v. 0–5) in a smoking subject, and alteration of gonadotropins (FSH: 36.8 mU/ml, n.v. 1.4–18.1; LH: 22.3 mU/mL, n.v.1.7–8.6) with low testosterone (1.13 ng/ml, n.v. 2.8–80) attributed to hypergonadotropic hypogonadism caused by bilateral cryptorchidism. Serum lactate, pyruvate, and thyroid hormones were normal, as were urinary organic acids. Immunologic evaluation showed only a slight reduction in lymphocytes (1.21 × 10^3^/μl, n.v. 1.50–3.50) and neutrophils (2.31 × 10^3^/μl, n.v. 2.50–7.50), with normal distribution of immunoglobulins IgG, IgM, and IgA.

Neurophysiological studies revealed an axonal sensory neuropathy, predominantly in the lower limbs. A brain MRI showed mild atrophy of the cerebellar vermis. A muscle CT scan showed increasing adipose muscle substitution. A muscle needle biopsy was performed in the left quadriceps, and showed only type 2 fibers hypotrophy. Neurophthalmological assessment showed normal fundi, but defective oculomotor control (nystagmus). Cardiac assessment confirmed a dilating cardiomyopathy (EF = 35%). Electroencephalography (EEG) showed slow basal activity with slow left temporal theta–delta waves, but no epileptic elements.

Neuropsychological assessment confirmed multiple-domain impairment with relative sparing of language, consisting of severe impairment of attention, visual perception, and verbal fluency by phonetic or semantic stimuli, deficient short-term verbal and visuo-spatial memory, and constructive apraxia.

#### II-2

II-2 showed cryptorchidism, low stature, hypotelorism, and short limbs. From 48 years of age, he reported progressive gait impairment, episodic neck and back pain, lower limb weakness, episodes of dizziness, and depression. He did not report speech difficulties. Multinodular thyroid hypertrophy was also present.

Neurological assessment reported horizontal nystagmus, impairment of eye pursuits, pes cavus, dysarthria, dysmetria, diffuse pyramidal signs, and ataxic and wide-based, mildly camptocormic gait.

As also seen in his twin brother, routine blood tests did not show alterations besides slightly elevated blood glucose (114 mg/dl) and increase in total cholesterol (285 mg/dl). Blood levels of gonadotropines were elevated (FSH: 27.4 mU/ml; LH*:* 16.9 mU/ml), and testosterone was low (0.84 ng/ml). Serum lactate, pyruvate, alpha-fetoprotein, and thyroid hormones were normal. The profile of urinary organic acids was normal as well. Immunologic evaluation showed a slight reduction in total leukocytes (3.88 × 10^3^/μl, n.v. 4.0–10.0), due to a decrease in neutrophils (1.69 × 10^3^/μl, n.v. 2.50–7.50); IgG, IgM, and IgA levels were normal.

Neurophysiological studies revealed axonal sensory neuropathy, with altered somatosensitive, motor, and brainstem auditory evoked potentials. Visual evoked potentials and electroretinogram were normal. Spinal and cerebral MRI was normal, with the exception of mild atrophy of the cerebellar vermis. EEG did not show epileptic elements. Cardiac assessment (ECG, echocardiogram and medical examination) revealed an initial left ventricular diastolic defect (EF = 52%). A muscle needle biopsy was performed in the left quadriceps, and showed only type 2 fibers hypotrophy.

Neuropsychological assessment revealed severe attention deficit with constructive apraxia, slow visual pursuit movements, difficulties in lexical production after phonemic stimulation, and long- and short-term visuo-spatial memory deficit.

## Results

Informed consent for genetic and biochemical studies, in agreement with the Declaration of Helsinki, was signed by the patients and healthy family members participating in this study.

### Genetic studies

Karyotype and array CGH analyses did not detect any alteration; mutations in *TIM14* and *FRDA* were excluded by direct sequencing in II-1. The analysis by both a forensic kit for genotyping and homozygosity mapping unequivocally showed that the two affected brothers are monozygotic twins.

We performed whole-exome sequencing on one proband (II-1) and his unaffected sister (II-3). First, we filtered out common variants, with a frequency> 0.1% in public databases, including dbSNPs, 1,000 Genomes, and Exome Variant Server. Then, based on a hypothesized recessive trait and on the known parental consanguinity, we selected non-synonymous variants in coding regions and splice-site junctions that were homozygous in II-1 and absent or heterozygous in II-3. The remaining variants were prioritized according to the predicted deleterious outcome of the corresponding amino acid substitutions (Supplementary Table S1). The most severe variant was the nucleotide change c.673C>T in *XRCC4*. Two different isoforms of *XRCC4,* 334 and 336 amino acid (aa) long, respectively, are produced by alternative splicing of the last exon, but the identified change is predicted to create in both a stop codon (p.R225*) with loss of one-third of the protein at the C-terminus. This portion of the protein contains a low-complexity domain with several sites that can be phosphorylated by DNA-PK, causing loss of DNA end bridging (Mahaney *et al*, 2013), whereas the head domain (aa 1–115) forming the hydrophobic core, and the stalk (aa 115–203), a coiled-coil domain important for dimerization and interaction with LIG4, are upstream the truncating mutation (Fig1B). A form of XRCC4 truncated for most of its C-terminus (aa 1–250) was reported to be able to complement the radiosensitivity of *XRCC4*-deficient cells (Koch *et al*, 2004). The variant was validated in affected patients (II-1 and II-2) by Sanger' sequencing and segregated within the family, being heterozygous in both father (I-1) and healthy sister (II-3) (Fig1C). The DNA from the mother was not available.

### *XRCC4* transcript and protein analysis

In order to evaluate the effect of the identified variant on the *XRCC4* transcript, we performed quantitative PCR on cDNA obtained from mRNA extracted from II-1, II-2, and control fibroblasts. We found that *XRCC4* transcript level was strongly reduced in mutant cells (Fig1D), probably due to nonsense-mediated mRNA decay.

Likewise, Western blot analysis showed undetectable XRCC4 protein in total lysates obtained from patients' fibroblasts by using two different polyclonal antibodies against XRCC4 (Fig1E). One antibody (Abcam) obtained using the full-length protein as antigen clearly recognized an *in vitro*-translated construct corresponding to the truncated XRCC4 protein; however, no signal was detected in patients' samples, demonstrating undetectable levels of both wild-type (wt) and mutant protein (Fig1D, Supplementary Fig S1). Immunofluorescence studies supported this result, showing the absence of any nuclear signal in patients' cells in contrast to the labeling observed in control cells (Supplementary Fig S2).

Notably, Western blot analysis revealed that, albeit reduced in amount (40% of control mean), LIG4 was clearly present in lysates from patients' fibroblasts (Fig1E).

### Functional validation in mutant fibroblasts

**Figure 2:**
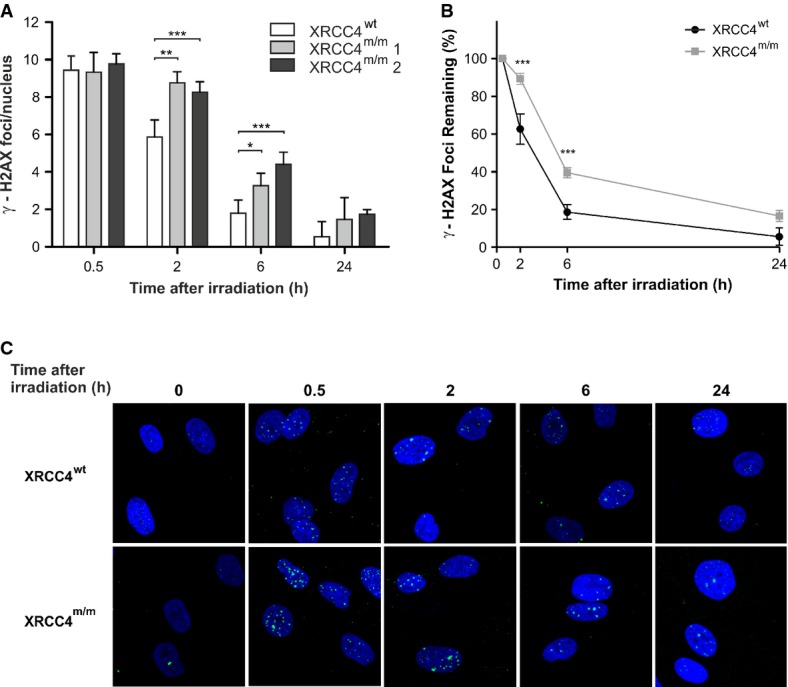
Analysis of γ-H2AX nuclear foci

To test the consequence of the *XRCC4* mutation on DNA repair efficiency, wt and mutant fibroblasts were first exposed to γ-rays (0.5 Gy), in order to induce DNA DSBs. After exposure to IR, the extensive phosphorylation at Ser139 of histone H2AX results in the formation of discrete γ-H2AX foci, which can be identified by immunostaining and constitute a useful tool to highlight the presence of DSBs (Fernandez-Capetillo *et al*, 2004; Sharma *et al*, 2012). Because this phosphorylation is abundant, fast, and correlates well with each DSB, it can be used to examine the DNA damage produced by irradiation and the subsequent repair of the DNA lesion (Svetlova *et al*, 2010). After irradiation, DSB rejoining was monitored for 24 h, by measuring the formation of γ-H2AX nuclear foci, as a DNA damage index, and their disappearance, as an index of DNA repair. During the 24-h monitoring, the decrease in foci number was significantly slower in *XRCC4*-mutant fibroblasts (*XRCC4*^*m/m*^ #1, from individual II-1; *XRCC4*^*m/m*^ #2, from individual II-2) compared to that in wt cells, whereas at the endpoint, the differences became statistically non-significant (Fig2A). Since the two *XRCC4*-mutant cell lines showed no significant differences in DSB rejoining, these data were pooled and expressed as a mean in subsequent analyses. The kinetics of γ-H2AX foci disappearance (Fig2B) confirmed that in *XRCC4*-mutant cells, the DNA lesions were repaired, although with lower efficiency than in wt cells, as the percentage of foci remaining at 24 h was 5 ± 8% in *XRCC4*^*wt*^ cells and 19 ± 7% in *XRCC4*^*m/m*^. This result suggests that undetectable levels of XRCC4 protein do not completely abolish DNA repair capability.

γ-H2AX nuclear foci in *XRCC4*^*wt*^ and mutant fibroblasts (*XRCC4*^*m/m*^1 and *XRCC4*^*m/m*^2) were determined at different times after irradiation with 0.5 Gy of γ-rays. Values, subtracted of their non-irradiated control (1.3 foci/nucleus in *XRCC4*^*wt*^ cells and 1.4 and 0.9 foci/nucleus in *XRCC4*^*m/m*^1 and *XRCC4*^*m/m*^2 cells, respectively), are the mean ± SD of three independent experiments.

Percentages of γ-H2AX foci in *XRCC4*^*wt*^ and *XRCC4*^*m/m*^ fibroblasts (pooled values) remaining at the indicated time-points.

Immunofluorescence micrographs of γ-H2AX foci in *XRCC4*^*wt*^ and *XRCC4*^*m/m*^ fibroblasts. Data information: ***P < 0.001; **P < 0.01; *P < 0.05 (*XRCC4*^*m/m*^ vs. *XRCC4*^*wt*^ cells), two-way ANOVA, Bonferroni *post hoc* test.

**Figure 3:**
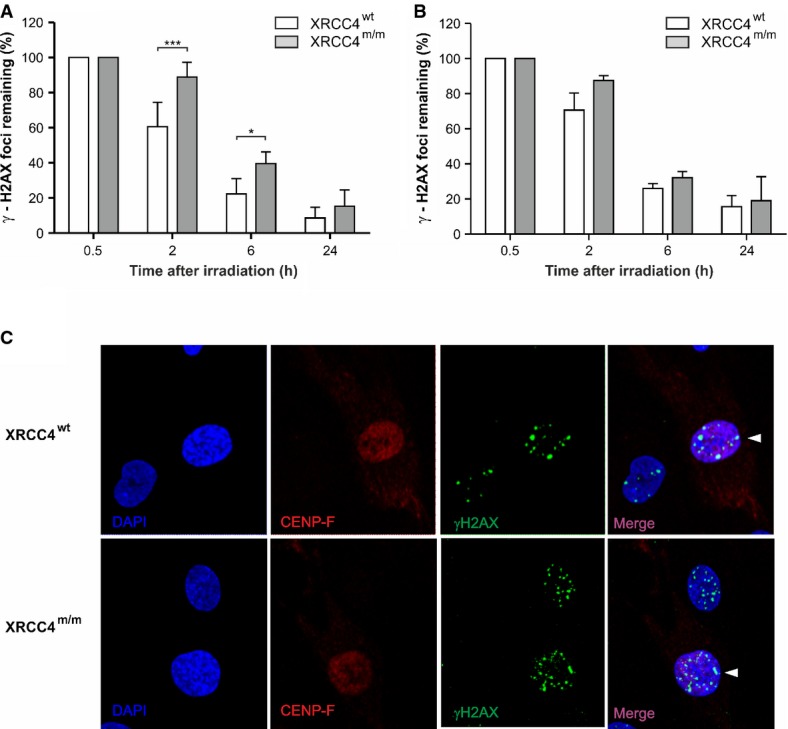
γ-H2AX nuclear foci formation in different cell cycle phases

To analyze the contribution of the two main DSB repair systems, NHEJ and HR, during cell cycle, we distinguished G1, S, and G2/M cells on the basis of their nuclear immunofluorescence intensity to the CENP-F protein, whose expression and localization are cell cycle dependent. CENP-F is detectable by *in situ* immunofluorescence throughout late S, G2, and M phases of the cell cycle, but it is absent in G1 (Bee *et al*, 2013). By staining the cells with a CENP-F antibody, we observed that in CENP-F-negative cells (G1-phase), in which DSB rejoining is carried out by NHEJ, DNA repair proceeded significantly slower in *XRCC4*^*m/m*^ than in *XRCC4*^*wt*^ cells (Fig3A), although at the 24-h endpoint, the differences became non-significant. In CENP-F-positive cells (late S and G2 phase), in which HR system is fully active, DSBs repair was similar at any time-point between wt and *XRCC4*-mutant cells (Fig3B).

A, B Percentages of γ-H2AX nuclear foci remaining in CENP-F-negative (A) and CENP-F-positive (B) cells at the indicated time-points after irradiation with 0.5 Gy of γ-rays. Values, subtracted of their non-irradiated controls, are the mean ± SD of three independent experiments. ***P < 0.001; *P < 0.05 (*XRCC4*^*m/m*^ versus *XRCC4*^*wt*^ cells), two-way ANOVA, Bonferroni *post hoc* test.

C Immunofluorescence micrographs of γ-H2AX foci in CENP-F-positive and negative fibroblasts at 2 h after irradiation. Arrow heads indicate the CENP-F-positive cells.

**Figure 4:**
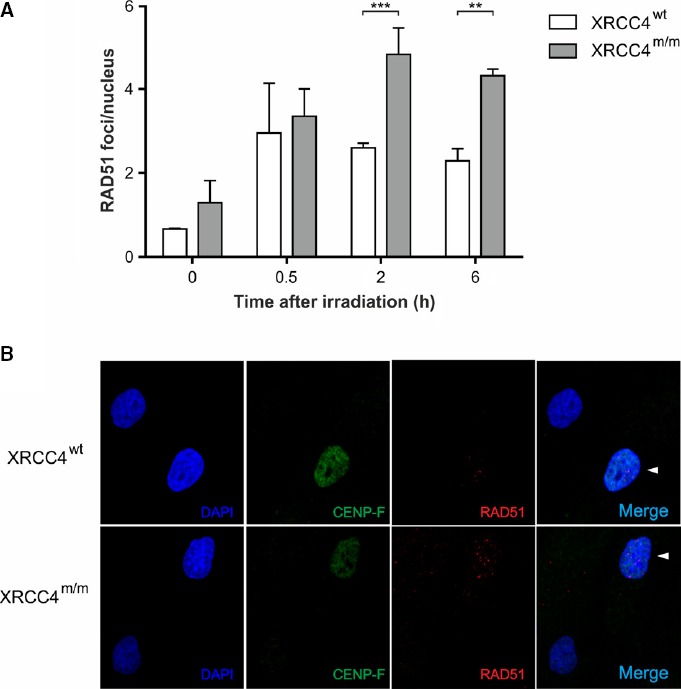
Involvement of homologous recombinations in repairing DNA DSBs

Next, we compared the involvement of HR in repairing DNA DSBs of *XRCC4*^*m/m*^ and *XRCC4*^*wt*^ fibroblasts, by analyzing formation and disappearance during the G2 phase of nuclear foci of RAD51, the central protein in the HR process (Fig4). The number of RAD51 foci at 2 and 6 h in *XRCC4*^*m/m*^ cells was significantly higher than in *XRCC4*^*wt*^ cells. This result suggests ‘compensatory' activation of HR in mutant NHEJ-defective compared to wt cells, where, as normally observed in higher eukaryotes, NHEJ is the main repair system active throughout the entire cell cycle. We could not reliably measure the number of RAD51 foci at 24-h post-irradiation endpoint because by then, only very few cells were in G2 phase (Table1) and the number of RAD51 foci/nucleus was negligible. Our data show that in *XRCC4*^*m/m*^ cells, the recruitment of HR factors at DSB sites significantly increased compared to *XRCC4*^*wt*^ cells, partially supplying NHEJ impairment.

**Table 1:** Cell cycle distribution (percentage ± SD)

IR	*XRCC4* ^*wt*^			*XRCC4* ^*m/m*^		
	G1	S	G2/M	G1	S	G2/M
−	88.62 ± 1.86%	4.97 ± 0.58%	6.42 ± 0.35%	92.23 ± 0.05%	2.52 ± 0.43%	5.25 ± 1.47%
+^a^	84.54 ± 2.90%	4.47 ± 0.67%	11.09 ± 0.80%	93.91 ± 0.33%	0.65 ± 0.21%	5.44** ± 0.77%

a Cell cycle analysis has been performed 24 h after irradiation (IR).

** *P* < 0.01 G2/M percentage in mutant fibroblasts versus G2/M percentage in wt fibroblasts, two-way ANOVA, Bonferroni *post hoc* test.

RAD51 foci in *XRCC4*^*wt*^ and *XRCC4*^*m/m*^ fibroblasts irradiated with 0.5 Gy of γ-rays. Values are the mean ± SD of three independent experiments. ***P < 0.001; **P < 0.01 (*XRCC4*^*m/m*^ versus *XRCC4*^*wt*^ cells), two-way ANOVA, Bonferroni *post hoc* test.

Immunofluorescence micrographs of RAD51 foci in *XRCC4*^*wt*^ and *XRCC4*^*m/m*^ fibroblasts. Arrowheads indicate the CENP-F-positive cells.

**Figure 5:**
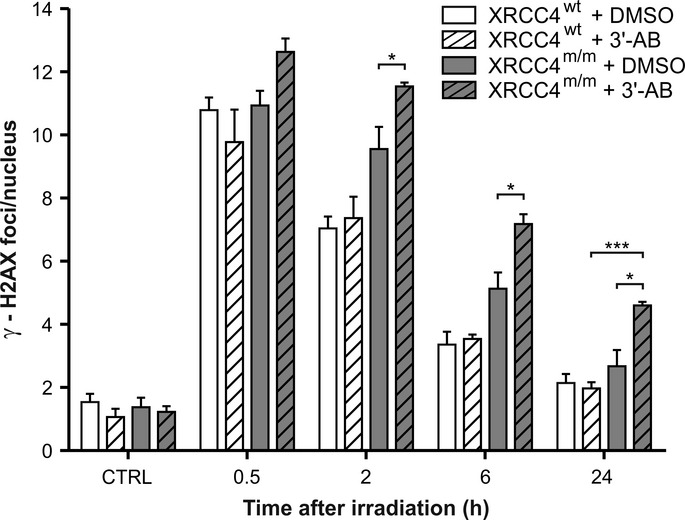
Analysis of PARP-1 inhibition on DSB repair *XRCC4*^*wt*^ and *XRCC4*^*m/m*^ cells irradiated with 0.5 Gy of γ-rays were exposed to the PARP1 inhibitor 3′-AB or to solvent (DMSO). The γ-H2AX foci number is the mean ± SD of three independent experiments. *P < 0.05 (*XRCC4*^*m/m*^ + 3′-AB versus XRCC4^m/m^ + DMSO), ***P < 0.001 (*XRCC4*^*m/m*^ + 3′-AB versus *XRCC4*^*wt*^ + 3′-AB), two-way ANOVA, Bonferroni *post hoc* test.

The fraction of cycling cells in a population of skin fibroblasts is usually low. In our experimental conditions (Table1), most of the cells were in G1 phase, as detected by analyzing DNA content. Before irradiation, 88.62 ± 1.86% of *XRCC4*^*wt*^ fibroblasts was in G1 phase and 6.41 ± 0.35% in G2/M phases; 24 h after irradiation, G2 cells increased up to 11.09 ± 0.80%, probably because of G2-checkpoint activation. In *XRCC4*^*m/m*^ non-irradiated cells, the percentages of G1 phase was 92.23 ± 0.43% and that of G2/M phases 5.25 ± 1.47%. At 24 h after irradiation, *XRCC4*^*m/m*^ cells in G1 phase were 93.91 ± 0.33% and those in G2/M were unchanged (5.44 ± 0.77%), probably because of failure of G2 checkpoint activation. Thus, the increased recruitment of HR factors to DNA lesions detected in *XRCC4*^*m/m*^-mutant cells in G2 phase (RAD51 foci in Fig4), which was about 5% of the total, cannot explain the persistence of the DNA repair level measured in our experiments. We therefore analyzed the involvement of the alternative non-homologous end-joining (A-NHEJ) pathway in DSB repair. The nuclear enzyme PARP-1 is usually involved in SSB rejoining by BER or NER processes, but in NHEJ-defective cells, it has been shown to participate in DSB repair, as a component of A-NHEJ (Wang *et al*, 2006; Mansour *et al*, 2010; Mladenov & Iliakis, 2011). To calculate the residual DSB rejoining upon inhibition of the A-NHEJ system, we treated *XRCC4*^*wt*^ and *XRCC4*^*m/m*^ fibroblasts with a PARP-1 inhibitor, 3′-aminobenzamide (3′-AB), 24 h before irradiation (Fig5). From 2 h after irradiation, the number of γ-H2AX foci in 3′-AB treated *XRCC4*^*m/m*^ cells was significantly higher than in untreated *XRCC4*^*m/m*^ cells. Conversely, in *XRCC4*^*wt*^ cells, where DSB repair is performed by the classical NHEJ (C-NHEJ) pathway, 3′-AB treatment did not modify the number of γ-H2AX foci found in untreated cells. We observed no difference in the amount of PARP-1 by Western blot analysis of *XRCC4*^*m/m*^ vs. *XRCC4*^*wt*^ cells, possibly because of qualitative changes in distribution (e.g. relocation of the protein within cell compartments) rather than quantitative variations in content.

**Figure 6:**
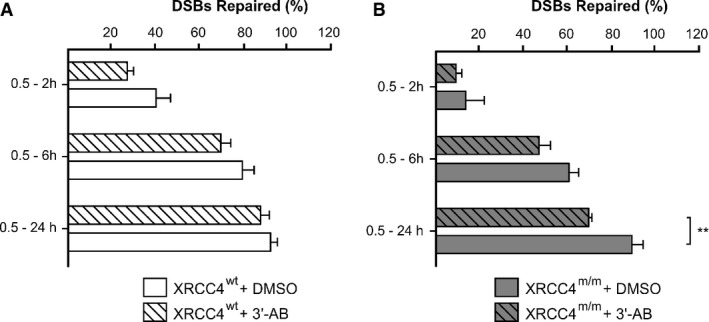
Quantification of alternative non-homologous end-joining (A-NHEJ) activity

From the number of γ-H2AX foci in presence or absence of PARP-1 inhibitor (Fig6), we can roughly estimate the percentage of DSBs induced by γ-ray irradiation that were rejoined by the A-NHEJ.

A, B Percentages of DSBs repaired in increasing time intervals (0.5–2 h, 0.5–6 h, 0.5–24 h) were calculated in *XRCC4*^*wt*^ (A) and *XRCC4*^*m/m*^ (B) cells from the number of γ-H2AX foci in presence or absence of a PARP-1 inhibitor, 3′-AB. **P < 0.01 (*XRCC4*^*m/m*^ + 3′-AB versus *XRCC4*^*m/m*^ + DMSO), two-way ANOVA, Bonferroni *post hoc* test.

Albeit indirectly, our data suggested that the involvement of A-NHEJ factors in repairing DSBs was irrelevant in XRCC4^wt^ cells (5 ± 3% of DSBs repaired within 24 h), while it was clearly significant in *XRCC4*^*m/m*^ cells (19 ± 4%).

## Discussion

The homozygous non-sense mutation in X-ray cross complementing gene 4 (*XRCC4*) found in our patients is predicted to result in the premature truncation of the XRCC4 protein, which was in fact undetectable in cultured mutant fibroblasts from both subjects. XRCC4 forms a complex with XRCC4-like factors, XLF/Cernunnos and PAXX, and DNA ligase IV (LIG4) (Critchlow *et al*, 1997; Grawunder *et al*, 1998a,b; Ahnesorg *et al*, 2006; Buck *et al*, 2006; Ochi *et al*, 2015), which operates the ligation step of NHEJ. Experimental evidence indicated that perturbations of the XRCC4–XLF interaction result in DSB repair deficits, including reduced frequency of coding end joining during V(D)J recombination. These observations suggested that, besides their well-characterized role in ligation step, XRCC4 and XLF may have multiple functions during NHEJ, including during early phases of the process, which may be independent from LIG4 (Roy *et al*, 2012). The newly discovered component PAXX functions with XRCC4 and XLF to mediate DSBR in response to DSB-inducing agents, promoting assembly and/or stability of the NHEJ machinery at DSB sites (Ochi *et al*, 2015). NHEJ is central to DNA DSB repair mechanisms but also to the mechanism operating the clonal somatic DNA shuffling responsible for the generation of the hyper-variable regions in antibodies and T-cell receptors (Helmink & Sleckman, 2012; Malu *et al*, 2012). Consequently, disruption of NHEJ proteins (Ku, DNA-PKcs, XLF, LIG4, and Artemis) often results in defects in DSB repair, radiation sensitivity, and severe combined immunodeficiency (SCID) (Li *et al*, 1995; Gu *et al*, 1997; Moshous *et al*, 2003; Buck *et al*, 2006). For instance, the LIG4 syndrome (OMIM #606593), caused by mutations in *LIG4*, is characterized by immunodeficiency, development and growth delay, unusual facial features, and microcephaly. Accordingly, cells from human patients with hypomorphic mutations in *LIG4* are radiosensitive, show impaired DSB repair, and display significantly elevated chromosomal breaks upon IR exposures (Riballo *et al*, 2001). Likewise, the NHEJ1 syndrome (OMIM #611291), caused by mutation in *XLF*, consists of severe combined immunodeficiency (SCID), microcephaly, growth retardation, and sensitivity to IR.

However, neither immunologic abnormalities nor susceptibility to cancer were part of the clinical phenotype of our *XRCC4*-mutant patients. Similar observations have been performed in cells derived from a patient with hypomorphic mutations in LIG4 that led to pronounced radiosensitivity but did not cause any major immune dysfunction, indicating that defects of DSBs repair are not necessarily associated with immune dysfunction. (Riballo *et al*, 1999). A homozygous missense variant in *XRCC4* was recently identified in a Saudi patient with primordial dwarfism, but it was not possible to establish its causative role because of the absence of any experimental validation (Shaheen *et al*, 2014). While this paper was under reviewing, two publications have reported patients with proven mutations in *XRCC4,* associated with primordial dwarfism (Murray *et al*, 2015) or early-onset metabolic syndrome (de Bruin *et al*, 2015). Despite the presence at birth of hypotelorism, short limbs, and short stature, our patients were not diagnosed as having primordial dwarfism and the main clinical feature was an adult-onset progressive encephalocardiomyopathy. They presented also cryptorchidism, suggestive of gonadal failure, which has been found in other *XRCC4*-mutant patients (de Bruin *et al*, 2015; Murray *et al*, 2015). In only one patient (P2 in de Bruin *et al*, 2015), a malignant tumor was reported, indicating that XRCC4 impairment is not strongly associated with predisposition to malignancy. Taken together, these reports, including our own, are concordant in showing that, quite unexpectedly, mutations in XRCC4, a crucial component of the NHEJ machinery, are not associated with immunodeficiency in humans.

In mice, genetic knockout (KO) of *XRCC4*, as well as *LIG4*, leads to embryonic lethality in conjunction with massive apoptotic death of neuronal cells in the nervous system (Frank *et al*, 1998; Gao *et al*, 1998; Chistiakov *et al*, 2009). Based on this observation in mice, human *XRCC4* mutations associated with primordial dwarfism have been deemed as hypomorphic alleles (Murray *et al*, 2015). However, most of the identified *XRCC4* mutations are nonsense or frameshift changes, predicting the synthesis of truncated proteins. Although the persistence of small amounts of mutant protein retaining some residual activity cannot be excluded, neither we nor others were able to detect any trace of truncated XRCC4 species, at least in patients' fibroblasts. This does not exclude the possibility that, in other cell types, mRNA decay or stability of the truncated protein may be different and these cells may utilize the truncated protein more effectively.

In contrast with previous reports suggesting that LIG4 is unstable in the absence of its partner protein XRCC4 (Ghezraoui *et al*, 2014) and is strongly reduced in *XRCC4*-mutant patients (Murray *et al*, 2015), we showed that LIG4 is moderately reduced in fibroblasts from our patients; this evidence could explain their milder phenotype.

In our patients, undetectable levels of XRCC4 caused neither immunologic defects nor increased susceptibility to cancer, and were instead associated with a relatively mild syndrome, including moderately severe congenital malformations, and a slowly progressive, adult-onset combination of cardiac failure, due to dilating cardiomyopathy, and neurological impairment characterized by cerebellar failure, cognitive arrest/decline, and behavioral abnormalities. Both patients are now 50 years old, and, albeit under constant tutoring, they still carry on a largely autonomous daily lifestyle.

Interestingly, neurodegeneration with ataxic features has been associated with defects in factors involved in signaling of DNA DSB damage, for instance ataxia-teleangiectasia (A-T) syndrome (OMIM #208900), a complex condition characterized by ataxia, teleangiectasiae, immunologic deficit, and predisposition to malignancy, caused by *ATM* mutations; or ataxia-telangiectasia-like syndrome (OMIM #604391), a phenocopy of, but milder than, A-T syndrome, caused by *MRE11A* mutations. Mutations in *APTX*, encoding aprataxin, cause autosomal recessive ataxia-oculomotor apraxia syndrome (OMIM #208920). Aprataxin seems to play an ill-defined role in regulation of NHEJ and detection of ssDNA breaks, by interacting with XRCC1, XRCC4 (Clements *et al*, 2004; Ahel *et al*, 2006), or PARP1 (Harris *et al*, 2009). In addition, defects in the repair of ssDNA breaks also cause neurodegenerative diseases such as spino-cerebellar ataxia with axonal neuropathy 1, SCAN1 (OMIM #607251), caused by mutations in tyrosyl-DNA phosphodiesterase 1 (*TDP1*), and microcephaly, seizures and developmental delay, MCSZ syndrome (OMIM #613402), caused by mutations in polynucleotide kinase phosphatase (*PNKP*). Notably, both SCAN1 and MCSZ show neither immunodeficiency nor cancer susceptibility. Thus, preferential involvement of the brain, particularly the cerebellum, is an intriguing feature of many disorders caused by defects of DNA repair pathways, including the very one presented here. Since HR is active only in proliferating cells, it is reasonable to hypothesize that a defect in the constitutively active NHEJ pathway can preferentially reduce the efficiency of DNA DBS repair in non-proliferating, post-mitotic, highly specialized cells, such as neurons (and cardiomyocytes). This consideration, together with evidence that NHEJ efficiency declines with aging (Vyjayanti & Rao, 2006; Vaidya *et al*, 2014), may explain the predominantly neurological manifestations of defective DNA repair disorders, including those associated with abolition of XCCR4.

Our study on patient-derived *XRCC4*^*m/m*^ fibroblasts demonstrated that the efficiency of IR-induced DNA DSBs repair was significantly reduced, but not abolished, during a 24-h long monitoring, and at the 24^th^-h endpoint, it was very similar to that of *XRCC4*^*wt*^ cells. As increased HR activity of *XRCC4*^*m/m*^ fibroblasts during the cell cycle G2 phase involved a very small fraction of cell population, we hypothesized the activation of other compensatory mechanisms. An alternative end-joining pathway is reported to become active when classical NHEJ is chemically or genetically compromised, operating as backup, hence called “backup non-homologous end joining” (B-NHEJ) or “alternative NHEJ” (A-NHEJ) (Wang *et al*, 2006; Iliakis, 2009). In *in vitro* studies, genetic defects of the NHEJ core component are associated with increased radiosensitivity and decreased repair efficiency of IR-induced DSBs. Defects in DNA-PKcs, as observed in the tumor cell line M059J, which lacks the catalytic subunit of DNA-PK, impair the efficiency of DNA repair, particularly for a decrease in DSB rejoined by fast kinetics. However, in these cells the level of residual DNA damage measured after long repair times is reduced (by 50% or less) compared with that measured in DNA-PKcs-proficient M059K cells (DiBiase *et al*, 2000; Bee *et al*, 2013). Similar results were obtained using mutant cells deficient in Ku, LIG4, or other components of classical NHEJ (Kabotyanski *et al*, 1998). These results suggested that cells with compromised C-NHEJ are capable of rejoining DNA DSBs, albeit through a repair pathway operating with slow kinetics, by some vicarious DSB rejoining process. Several laboratories have identified factors participating in the alternative pathway of NHEJ: for instance, LIG3 (DNA ligase III; Simsek & Jasin, 2011), heretofore known only for its role in BER, was considered the candidate ligase required for A-NHEJ (Wang *et al*, 2005); PARP1 (poly ADP-ribose polymerase 1) and XRCC1 (X-ray cross complementing gene 1), two proteins known to interact with LIG3 during BER, were subsequently found to be involved in A-NHEJ (Audebert *et al*, 2004; Wang *et al*, 2006; Cheng *et al*, 2011). PARP1 is presumed to compete with Ku for binding to broken DNA ends, thereby dictating the pathway of choice (Wang *et al*, 2006; Cheng *et al*, 2011), whereas XRCC1 appears to act as a chaperone for LIG3 (Caldecott *et al*, 1994). The A-NHEJ is thought to operate with much lower fidelity than C-NHEJ and has been implicated in oncogenic genome rearrangements, mainly chromosomal translocations, both in cancer and in cultured cells (Bétermier *et al*, 2014). In our experiments, we demonstrated that the A-NHEJ is active only in *XRCC4*^*m/m*^ cells, whereas it did not contribute to the DNA end rejoining in *XRCC4*^*wt*^ cells. However, in mutant fibroblasts treated with the A-NHEJ inhibitor, 3′-AB, DNA repair capability was significantly decreased but not abolished, showing that, at least in those experimental conditions, *XRCC4*^*m/m*^ cells still retain the capability of rejoining more than 50% of DSBs induced by irradiation. Taken together, our findings suggest that some vicarious mechanisms, only partly relying on the activation of the A-NHEJ pathway, make the XRCC4 protein remarkably dispensable in humans, at least to warrant immunologic proficiency and anti-cancer surveillance.

## Materials and Methods

Informed consent for participation in this study was obtained from all investigated subjects, in agreement with the Declaration of Helsinki and approved by the Ethical Committee of the Foundation IRCCS (Istituto di Ricovero e Cura a Carattere Scientifico) Institute of Neurology “Carlo Besta”.

### Molecular studies

Total genomic DNA was extracted by standard methods from peripheral blood lymphocytes. Total RNA was isolated from cell pellets using the RNeasy Mini Kit (Qiagen) and reverse-transcribed to GoTaq® 2-Step RT-qPCR System (Promega), following manufacturer recommendations. *XRCC4* expression in cDNA samples obtained from DNase-treated mRNA was determined using reverse transcription quantitative PCR (qPCR) with SYBR Green chemistry and the following specific *XRCC4* primers:

207F: TGGACTGGGACAGTTTCTGA, 270R: TCAGTTCACCAACATATTTCCC;
387F: GATGTCTCATTCAGACTTGGTTCCT, 458R: AAGTTCTCTAATGACTTCAGCTGGG.

*GAPDH level was used for normalization*.

### Whole-exome sequencing

Whole-exome sequencing was performed on genomic DNA from II-1 and II-3 by “BGI-Shenzhen”. The DNA sample was randomly processed in fragments between 200 and 300 bp. In-solution targeted enrichment of exonic sequences was performed using the 64 Mb Exon Capture kit from Nimblgen. Then, the captured library was sequenced on Hiseq2000 platform (Illumina). GeneTalk (www.gene-talk.de) and Ingenuity Variant Analysis (www.ingenuity.com) web applications were used for variant filtering. Polyphen2, SIFT, and Mutation taster were used for pathogenicity prediction of amino acid changes.

### Cell cultures

Primary fibroblasts, obtained by standard protocols from skin biopsies from patients and control subjects, were cultured in high glucose DMEM (Euroclone) supplemented with 10% FBS/FCS, 1% L-glutamine, HEPES 20 mM, and 1% MEM non-essential amino acids, in a 37°C incubator with 5% CO_2_ and 100% humidity. All cells were tested for mycoplasma contamination. Analyses were performed at passages 3–7.

### Immunoblot analyses

Approximately 10^6^ cells from patients and controls were trypsinized, pelleted, and solubilized. SDS–polyacrylamide gel of 50 μg protein/lane and Western blot analysis were performed using antibodies against XRCC4 (Santa Cruz sc-8285; Abcam Ab145), PARP-1 (Roche), LIG4 (GenTex GTX108820), and GAPDH (Millipore) as loading control. Immune-visualization was carried out by chemiluminescence-based ECL kit (GE Healthcare). ^35^S-radiolabeled *in vitro*-translated products corresponding to full length and the R225* ORFs were obtained with TNT Quick Coupled Transcription/Translation Systems (Promega).

**The paper explained**

**Problem**

Two twins with an adult-onset encephalocardiomyopathy were found to carry a homozygous nonsense mutation in *XRCC4*. XRCC4 functions in non-homologous end-joining (NHEJ) repair of double-strand DNA breaks; inactivation of murine *Xrcc4* results in a lethal phenotype with widespread neural apoptosis.

**Results**

*XRCC4* transcript was profoundly reduced, and the protein was undetectable in patient tissues/cells. Functional assays on mutant cells demonstrated reduction, but not abolition, of the ability to repair double-strand DNA breaks. Accordingly, patients reached adulthood and have no sign of immunodeficiency or malignancy.

**Impact**

These results indicate redundant or compensatory mechanisms to the virtual absence of XRCC4 in humans. XRCC4 seems dispensable in humans, at least to warrant immunologic proficiency and anti-cancer surveillance.

### Cell treatments

Gamma irradiation was performed at the Department of Oncological and Surgical Sciences of the University Padova Medical Center with a ^137^Cs source (dose rate 2.8 Gy/min). Cells (20,000/sample) were seeded on 35-mm μ-Dish thin-bottomed Petri dishes for high-end microscopy (Ibidi) in random positions 48 h before irradiation and incubated in fresh medium for different repair times. When indicated, PARP-1 inhibitor (10 mM 3′-aminobenzamide in DMSO, Sigma-Aldrich) or 0.1% DMSO was added 24 h before irradiation and maintained throughout experiments.

### Immunofluorescence staining and foci counting

At 0.5, 2, 6 and 24 h after gamma irradiation, cells were rinsed with 1× PBS and fixed with 4% formaldehyde (Sigma-Aldrich) in PBS for 15 min at 37°C. Cells were then washed twice with PBS and permeabilized with 0.2% Triton X-100 in PBS at 37°C for 10 min. Non-specific binding sites were masked with horse serum (10% in PBS) for 1 h at room temperature. Samples were incubated for 2 h at room temperature with anti-γ-H2AX (Ser139) (Millipore, Clone JBW301, 1:100), anti-RAD51 (Santa Cruz Biotechnology, H-92: sc-8349, lot. G0811, 1:100), and anti-CENP-F (BD Bioscience, 610768, Clone 11, 1:100 or Abcam, ab5, lot. GR73067-3, 1:300) primary antibodies followed by three washings in PBS + 0.05% Tween-20. The cells were incubated for 1 h at room temperature with Alexa Fluor 488 goat anti-mouse and Alexa Fluor 594 donkey anti-rabbit secondary antibodies (1:250, Life Technologies), washed three times with PBS + 0.05% Tween-20 and once with milli-Q water. Coverslips were mounted on samples with Vectashield medium (Vector Laboratories) containing 0.2 μg/ml of DAPI. Samples were acquired by Z-plane stack scanning (500 nm thickness) using a Leica TCS SP5 confocal microscope (Leica Microsystems) equipped with 40× oil immersion objective. γ-H2AX and RAD51 foci were scored by eye by two different researchers from 100 nuclei for each time-point in at least three independent experiments. The nuclear fluorescence intensity of CENP-F protein was used to discriminate the γ-H2AX and RAD51 foci in the S-G2 and G1 cells as previously described (Bee *et al*, 2013).

### FACS analyses

Cell cycle distribution of irradiated and non-irradiated control cells was assessed in three independent experiments by flow cytometry analysis of DNA content following staining with 50 μg/μl of propidium iodide (Sigma-Aldrich), as previously described (Bee *et al*, 2013). Data were collected from 25,000 cells/sample using a BD FACSCanto™ II flow cytometer (Becton Dickinson) and analyzed by ModFit LT software (Verity Software House).

### Statistical analysis

Statistical analyses were performed using Primer of Biostatistics software (McGraw-Hill), and figures were prepared with Corel Draw X6 (Corel corporation). The variance, assessed by ANOVA test, was similar between the groups that were compared.

## For more information

http://omim.org/entry

http://www.biobanknetwork.org

http://www.mrc-mbu.cam.ac.uk

http://www.mitopedia.org

http://www.unipd.it/international-area/departments/departments/department-biology

## Supporting Information

Supplementary Table S1

Review Process File
