# Supplementary material for: A nonsense mutation of human XRCC4 is associated with adult-onset progressive encephalocardiomyopathy
Source: EMBO Mol Med. 2015 Apr 14;7(7):918–29. doi: 10.15252/emmm.201404803 (PMC4520657; doi:10.15252/emmm.201404803)
Supplement: Supplementary file 1 [file emmm0007-0918-sd1.pdf]

Figure S1

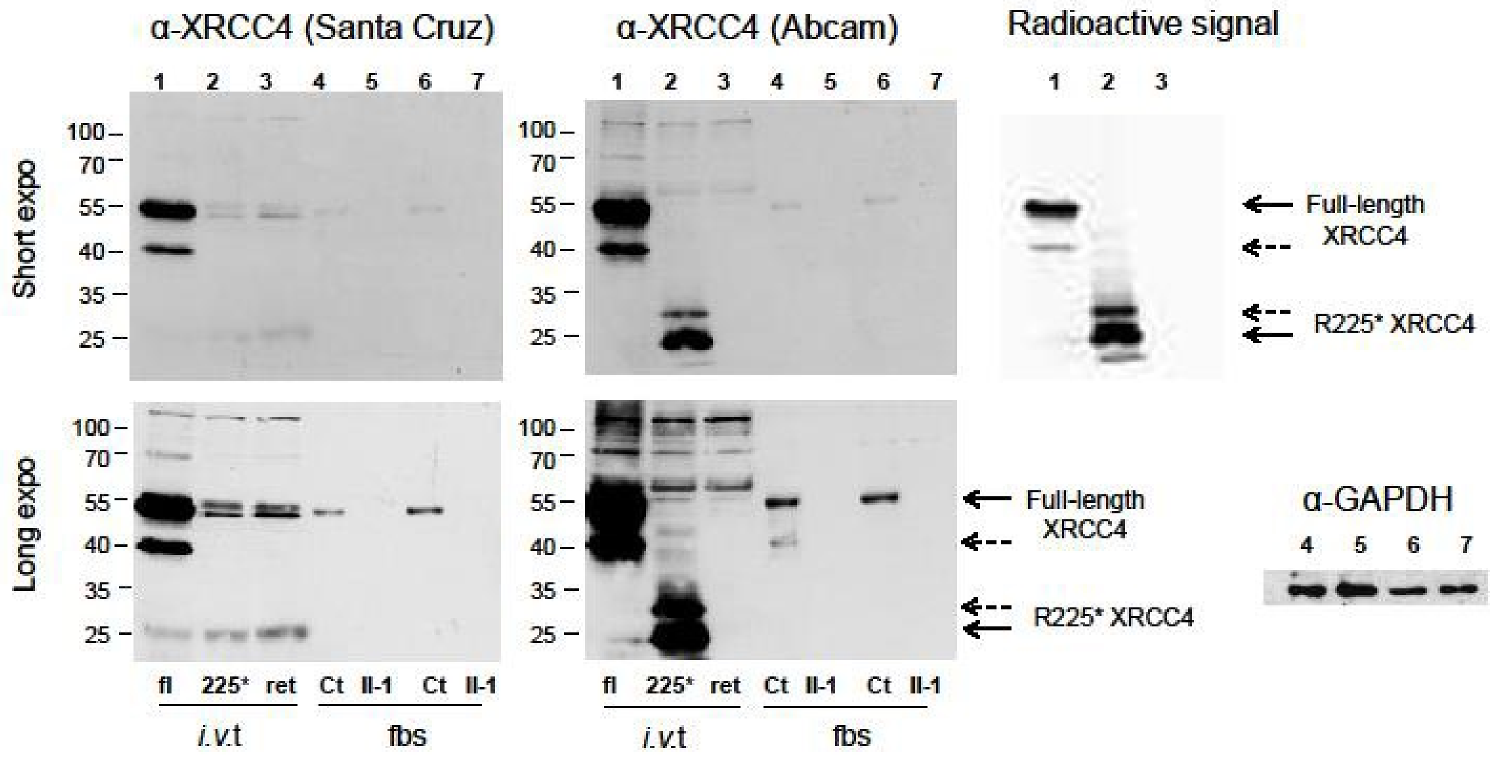


**Figure S1**. **Immunoblot analyses using anti-XRCC4 antibodies**

Immunoblot analysis of in vitro synthesized radiolabeled proteins (i.v.p) and total lysates from fibroblasts (fbs). fl: in-vitro translated full-length XRCC4 protein; 225*: in-vitro translated R225* truncated XRCC4 protein; Ret.: reticulocyte lysate used for in-vitro protein synthesis; Ct: lysate of control fbs; II-1: lysate of fbs from patient II-1. Two independent cell preparations were loaded.

The position of the molecular weight (MW) marker proteins is indicated. GAPDH was used as loading control. The radioactive signals of i.v.p were used to confirm the specificity of the bands detected by the polyclonal antibodies against XRCC4.

## Figure S2

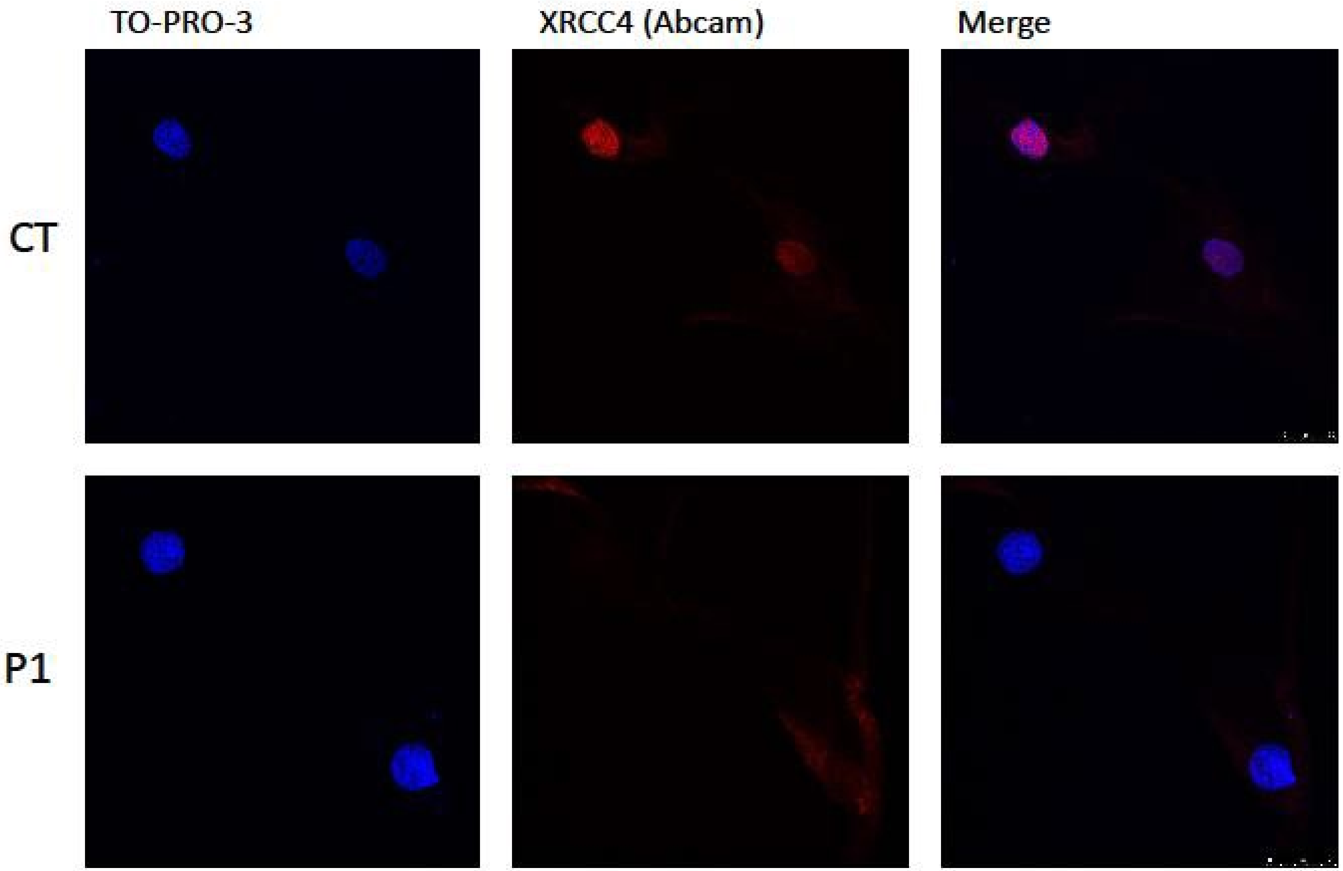


**Figure S2**. **Immunofluorescence studies**

Representative images of control (Ct) and patient (II-1) fibroblasts; cells were stained using α-XRCC4 antibody (Abcam) and To-Pro-3 (Lifesciences), a nuclear dye. Scale bars represent 25 µm.
