# Supplementary material for: A nonsense mutation of human XRCC4 is associated with adult-onset progressive encephalocardiomyopathy
Source: EMBO Mol Med. 2015 Apr 14;7(7):918–29. doi: 10.15252/emmm.201404803 (PMC4520657; doi:10.15252/emmm.201404803)
Supplement: Supplementary file 2 [file emmm0007-0918-sd2.pdf]

A nonsense mutation of human XRCC4 is associated with adult-onset progressive encephalocardiomyopathy

Leonardo Bee et al.

Supplementary information

Supplementary information includes 1 Table and 2 Figures

Supplementary Table S1

Chr	Position	Ref	Alt	Genes	Transcript	cDNA	Protein	SIFT	PolyPhen2	Taster	Score*	EVS %
5	82500668	C	T	XRCC4	NM_003401	c.673C>T	p.R225X	Damaging	Damaging	disease	**3**	0,00
16	11935849	C	G	RSL1D1	NM_015659	c.644G>C	p.R215P	Damaging	Possibly dam	disease	2,5	0,00
19	10132007	T	A	RDH8	NM_015725	c.613T>A	p.F225I	Damaging	Possibly dam	disease	2,5	0,00
19	9492071	T	G	ZNF177	NM_001172651	c.584T>G	p.L195R	Damaging	Probably dam	polim	2	0,02
19	10475315	G	A	TYK2	NM_003331	c.1342C>T	p.R448W	Damaging	Possibly dam	polim	1,5	0,01
5	139715520	G	A	HBEGF	NM_001945	c.491C>T	p.A164V	Tolerated	Benign	disease	1	0,00
19	36368996	T	A	APLP1	NM_001024807	c.1562T>A	p.M521K	Activating	Benign	polim	1	0,03
X	9679796	G	A	TBL1X	NM_001139468	c.1585G>A	p.V529I	Tolerated	Benign	disease	1	0,00
5	81549231	G	A	ATG10	NM_031482	c.650G>A	p.R217Q	Tolerated	Benign	polim	0	0,00
X	106486493	A	G	PIH1D3	NM_173494	c.610A>G	p.M204V	Tolerated	Benign	polim	0	0,00

* Arbitrary score from the prediction softwares (Damaging, Disease=1; Probably or possibly damaging=0.5; Benign, tolerated=0)
