# Supplementary material for: A nonsense mutation of human XRCC4 is associated with adult-onset progressive encephalocardiomyopathy
Source: EMBO Mol Med. 2015 Apr 14;7(7):918–29. doi: 10.15252/emmm.201404803 (PMC4520657; doi:10.15252/emmm.201404803)
Supplement: Supplementary file 3 [file emmm0007-0918-sd3.pdf]

A nonsense mutation of human XRCC4 is associated with adult-onset progressive encephalocardiomyopathy

Leonardo Bee, Alessia Nasca, Alice Zanolini, Filippo Cendron, Pio D'Adamo, Rodolfo Costa, Costanza Lamperti, Luciana Celotti, Daniele Ghezzi and Massimo Zeviani

Corresponding authors: Daniele Ghezzi, Foundation IRCCS Institute of Neurology "Carlo Besta" and Massimo Zeviani, Foundation IRCCS Institute of Neurology and MRC Mitochondrial Biology Unit

Review timeline:

Submission date:	31 October 2014
Editorial Decision:	21 November 2015
Revision received:	28 February 2015
Additional author correspondence	13 March 2015
Editorial Decision:	14 March 2015
Revision received:	20 March 2015
Editorial Decision:	23 March 2015
Revision received:	23 March 2015
Accepted:	24 March 2015

Transaction Report:

(Note: With the exception of the correction of typographical or spelling errors that could be a source of ambiguity, letters and reports are not edited. The original formatting of letters and referee reports may not be reflected in this compilation.)

Editor: Céline Carret

1st Editorial Decision 21 November 2015

Thank you for the submission of your manuscript to EMBO Molecular Medicine. We have now heard back from the three referees whom we asked to evaluate your manuscript. Although the referees find the study to be of potential interest, they also raise a number of concerns that must be addressed in the next version of your article.

As you will see from the comments pasted below, the three referees find the study clinically interesting. However, due to the unexpected phenotype, they also do require further experiments to improve conclusiveness and better substantiate the conclusions, especially as XRCC4 KO in human cells do not recapitulate these findings. For example, we would strongly encourage you to investigate the nature of the XRCC4 mutation *in vitro* as suggested by the referees, as well as whether residual activity do exist. In addition, experiments to further knock down XRCC4 or rescue its expression would certainly shed light into the molecular mechanism. Finally, the text should be tighten and refocused as suggested.

Given these evaluations, I would like to give you the opportunity to revise your manuscript, with the understanding that the referee concerns must be fully addressed and that acceptance of the manuscript would entail a second round of review. Please note that that it is our journal's policy to allow only a single round of revision, and that acceptance or rejection of the manuscript will therefore depend on the completeness of your response and the satisfaction of the referees with it.

EMBO Molecular Medicine has a "scooping protection" policy, whereby similar findings that are published by others during review or revision are not a criterion for rejection. Should you decide to submit a revised version, I do ask that you get in touch after three months if you have not completed it, to update us on the status.

Please also contact us as soon as possible if similar work is published elsewhere. If other work is published we may not be able to extend the revision period beyond three months.

I look forward to receiving your revised manuscript.

***** Reviewer's comments *****

Referee #1 (Comments on Novelty/Model System):

It would give additional info if the mutant XRCC4 protein was studied in vitro to determine whether there is residual activity.

Referee #1 (Remarks):

Bee et al. describe in two monozygotic twins born from consanguineous parents an late-onset (adult-onset) of progressive encephalocardiomyopathy. Using WES they identified a homozygous nonsense mutation in the XRCC4 gene. The authors claim that this is the first XRCC4 mutation described in human. However, earlier this year, Shaheen et al. published in Genome Research a patient with primordial dwarfism due to a mutation in XRCC4 (NM_003401.3: c.127T>C, p.Trp43Arg). This paper should be referred and discussed in the current manuscript. The clinical description is still highly relevant, but it is not the first XRCC4 deficient patient. This manuscript gives a detailed study on the effects of the XRCC4 mutation on NHEJ and HR.

1. In its current form the case report are rather extensive. The authors might consider to shorten them.
2. Via RT-PCR and western blot analysis, a strongly reduced level of XRCC4 transcripts was detected, but protein could not be detected. Is this mutation regarded as a null mutant or can residual activity be expected? To address this point, the mutation needs to be cloned in an expression vector and activity needs to be measured. This would give additional info for correct interpretation of the mutation.
3. The authors did not find any immunological abnormalities in both patients, although immunodeficiency was expected based on the function of XRCC4. Could you speculate on how this could be explained e.g. redundancy or other mechanisms in the context of V(D)J recombination?
4. Have patients with similar clinical characteristics been tested for mutations in XRCC4? The paper would be strengthened if more patients could be included.

Minor points:
1. The readability of Figure 5 can be improved if the percentages are also given in the figure.

Referee #2 (Comments on Novelty/Model System):

Zeviani and coworkers use throughout the manuscript cells derived from two patients to draw most of their conclusions. The authors should complement these cells derived from the patients with the cDNA coding for XRCC4 to confirm that the observed phenotype is a direct consequence of the nonsense mutation found on XRCC4 in the two patients reported. Indeed they also report in the patients several additional homozygous mutations potentially deleterious that are not tested in the manuscript.

Referee #2 (Remarks):

Zeviani and co-workers report here the identification of the first human syndrome caused by a mutation in the XRCC4 gene creating a premature stop codon. The main known function for the

XRCC4 protein is to stabilize DNA ligase IV, a protein mediating the final ligation step of the main DNA repair pathway of DNA double-strand breaks (DSB): Non Homologous End Joining (NHEJ). Several other human syndromes have already been reported to be caused by mutations in proteins of the NHEJ repair machinery such as Artemis and XLF/Cernnunos. Inactivating mutations in NHEJ proteins usually result in radiosensitivity, cancer predisposition and severe combined immunodeficiency (SCID) due to the essential role of NHEJ in the processes of V(D)J recombination and class-switch. Strikingly, the two patients reported here do not display immunodeficiency nor radiosensitivity nor cancer predispositions but instead a progressive encephalocardiomyopathy. When Zeviani and co. performed cellular analysis of fibroblasts derived from these patients they observed only a moderate repair defects, in contrast to what have been reported for XLF deficient cells for example (Buck et al. Cell 2006; Ahnesorg et al. Cell 2006). The most striking observation was a change in the balance between the different DNA double-strand break repair pathways with a higher proportion of DNA double-strand breaks being repaired by homologous recombination and by an alternative, slower and PARP-dependant DSB repair pathway known as alt-NHEJ or b-NHEJ. While the findings presented here are novel and provocative, they are also in direct contradiction with a recent work describing a complete knock-out of XRCC4 in human cells (Ghezraoui et al. 2014 Mol Cell). Indeed a clear NHEJ-dependant DNA repair defect is observed in these cells (similar to DNA Ligase IV knock out). In addition, other approaches (siRNA or shRNA) have also previously shown that reducing the level of XRCC4 in human cells leads to a repair defect that correlates with the efficiency of the siRNA/shRNA. In fact, siRNA against XRCC4 are frequently used as a positive control in DNA repair assays (see for example Ahnesorg et al. Cell 2006; Meerang et al. Nat Cell Biol 2011).
Therefore, from the data presented, two hypotheses have to be tested to explain this discrepancy (the absence of a clear DNA repair defect):
- the mutation reported might not be fully inactivating but instead hypomorphic.
- or one of the other homozygous and potentially inactivating mutations identified might compensate for the inactivation of XRCC4
Provided that the experiments necessary to test both hypotheses are conclusively performed (see below), the manuscript would be suitable for publication in EMBO Molecular Medicine. The quality of some experiments should also be improved (cell cycle distribution analysis) to reach the standards of EMBO Mol Med.

Major comments:

- Despite the level of XRCC4 being strongly reduced as shown in Fig1, a truncated version of XRCC4 might still be expressed and the reported phenotypes would therefore result from the hypomorphic nature of the mutation. Indeed it has been reported that a form of XRCC4 truncated for most of its c-terminus (XRCC4 1-250) is still able to complement the radiosensitivity of XRCC4 deficient cells (Koch et al. EMBO J 2004; should be cited in the manuscript); and XRCC4 1-225 still contains the DNA ligase IV interaction region (aa 173-195). To confirm that XRCC4 is not expressed on the form of a fragment, a new XRCC4 blot should be provided showing smaller proteins, together with the position of molecular weight markers.

- More importantly, since the main known function for XRCC4 is the stabilization of the DNA ligase IV protein, it is crucial to check the level of DNA ligase IV in patient cells as compared to control cells. If the levels are unaffected, or moderately affected, this would support the hypomorphic nature of the mutation and explain the lack of immunodefficiency in these patients, Different labs have developed anti-DNA Ligase IV antibodies that can be requested to perform this crucial control.

- Since several additional homozygous and potentially inactivating mutations have also been discovered in these patients it is crucial to check that the main phenotypes reported here are due to the mutation on XRCC4, especially the reported imbalance between the DNA repair pathways. A rescue experiment in patient cells with the cDNA coding for human XRCC4 should be performed to check if the phenotypes observed in the patient cell lines (especially Rad51 foci and impact of the PARP inhibitor) are rescued by the re-expression of XRCC4. This is a standard experiment when attributing a phenotype to a mutation.

- knock-down experiments (siRNA/shRNA) should be performed on the patient cells to analyse if a further reduction of XRCC4 level (even if undetectable) would lead to an additional radiosensitivity. It has to be noted that knocking-down XRCC4 in human cells using siRNA have been used as a positive control in several works. In Ahnesorg et al. Cell 2006 for example, siRNA XRCC4 gives a sensitivity to ionizing radition similar to siRNA against XLF.

- Zeviani and co. also identified at least two other proteins carrying potentially inactivating mutations (RSL1D1 and RDH8) and at least one of them has a link with the DNA damage response (RSL1D1, that "acts as a pro-apoptotic regulator in response to DNA damage" (Uniprot)). These mutations might compensate for the loss of XRCC4. If the authors failed to show that the mutation reported in XRCC4 is hypomorphic (see above), they should try to complement the patient cells with the cDNA of RSL1D1 and RDH8 to test if a reexpression of the wild-type form of these proteins radiosensitise the mutant cells but not the wild type cells.

Minor comments:

- most experiments do not include a positive control, such as the use of another human cell line deficient for another NHEJ component. This would greatly help to appreciate the sensitivity of the assays used here (maybe the mild effect of the mutation on DNA repair is just related to a technical problem) and provide a point of comparison. XLF deficient cell lines have been described (Ahnesorg P et al. Cell 2006 and Buck et al. Cell 2006).

- the cell cycle analysis in Fig. 5 should be repeated with a more accurate quantification of S-phase index based on a specific staining of replicating cells (BrdU or EdU staining).

- sequence of primers used for RT-PCR should be provided

- to allow further studies to take place, authors should engage in establishing immortalized derivatives of the fibroblast cells used here.

- p12: Zeviani and co. discuss about the "Cernnunos syndrome" and give its OMIM ID as 611290. Both information are incorrect: the syndrome is named the "NHEJ1 syndrome" and its OMIM ID is 611291. The ID 611290 refers to the XLF gene itself in the OMIM database.

- in few instances inappropriate publications are cited. For example:
+ Riballo and al. 2009 did not show that XRCC4 interaction with DNA ligase IV is required to stabilize DNA ligase IV. Instead, this was reported in Bryans et al. Mutat Res 1999 (CHO cells) and later confirmed in human in several reports (e.g. Wu et al. J Biol Chem 2007).
+ Corneo et al. 2007 was not the first publication describing alternative NHEJ. This was reported in Wang et al. Nucleic Acids Research 2003 and other reports (Audebert et al. 2004).

Referee #3 (Comments on Novelty/Model System):

XRCC4-mutant patients are very novel, and cells from the patients are an appropriate model. Studies can be improved as discussed, and the medical impact is relevant to a small but growing subset of patients with DNA damage response defects.

Referee #3 (Remarks):

Bee et al. report intriguing findings in identical twins sharing a mild syndrome characterized by neurological, cardiac and developmental symptoms. Whole exome sequencing identified a clearly consequential homozygous mutation, consistent with consanguinity, leading to a premature stop codon in the XRCC4 gene involved in the nonhomologous end joining (NHEJ) DNA repair pathway. Remarkably, the patients did not show some expected phenotypes, in particular immunodeficiency, typically associated with NHEJ deficiency, and the cellular DSB repair defect was also relatively mild.

These findings are of potentially substantial interest to both human geneticists and DNA repair biologists. One the one hand, they might provide an explanation as to why XRCC4-deficient humans have not been identified among cohorts used to find patients with defects in the related LIG4 and XLF genes. However, it is those patients, and an extensive literature documenting the phenotypes of XRCC4-deficient cells, that make it challenging to understand the current results and patients. In general, it is plausible that the identified XRCC4 mutation is a major part of the cause of the patients' disease (although no other alleles are pursued). However, it is less certain that they have as complete of an XRCC4 deficiency as suggested. The truncation would leave a large portion of the XRCC4 protein intact, and in particular the portion that would likely support its continued dimerization and interactions with LIG4 and XLF. It would thus seem possible that this is a hypomorphic allele, in contrast to the complete loss of XRCC4 function suggested (even in the face of the potential for nonsense-mediated decay). In addition to some important text and other improvements, the paper would benefit from a more rigorous evaluation of the potential for residual XRCC4 expression and function.

1) The critical issue is whether there really is a complete absence of XRCC4 protein. If the protein is truly absent, then the results would represent a substantial departure from previously described XRCC4 and NHEJ biology. This possibility is real and not discounted, and could reveal an interesting facet of human-specific NHEJ biology, but such a claim requires a rigorous demonstration beyond what is supplied. The results will be of interest even if the allele proves to be hypomorphic, but would be generally more consistent with expectations.

A) The Western blot should be repeated and presented:
(i) in a manner that allows the reader to examine both the position of the wild-type protein and the predicted position of the mutant protein, i.e. the whole blot should be shown, with molecular weight markers.
(ii) at a substantially greater "over-exposure" to show potentially low level proteins.
(iii) with at least one other, and ideally a polyclonal, antibody to corroborate results and guard against unanticipated false negative results.
(iv) ideally with an in vitro generated protein of the form anticipated and/or some other source of XRCC4 protein to allow determination of the blot sensitivity (how much protein could be detected?).

B) There is a nearly complete absence of information presented as to the presumptive protein-level effects of any residual amount of mutant XRCC4 protein. This should be corrected, including the addition of a figure descriptive of the location of the mutation relative to the known protein domains. There is abundant information to consider that most notably includes crystallographic and functional biochemistry of wild-type and mutant proteins. This is not inconsequential, as there is an active recruitment of NHEJ repair proteins to DSBs, and even a minor "undetectable" amount of XRCC4 protein might be sufficient to supply a borderline NHEJ function consistent with the observations.

C) Although it moves beyond patient cells, it might be informative to establish a human XRCC4 knock-out/down cell system and ask whether the mutant allele could complement observed phenotypes.

2) It is standard practice in DNA repair literature to show example photographic images of cell foci (H2AX and Rad51) to allow the reader to judge the quality and nature of results.

3) The DNA content figure is not particularly interesting or informative.

4) The inclusion of the a-NHEJ-directed experiments is good and worthwhile, but at points results are over-stated with respect to the nature and precision of the manipulation and its effect on a-NHEJ. 3-AB is a reasonable experiment, but cannot be considered a highly targeted impairment of a-NHEJ.

5) Unlike most of the paper, the Introduction is poorly presented. It includes a large amount of irrelevant or misplaced information and lacks many topics of relevance to understanding the Results that follow. There are additionally some errors.

A) The detailed discussion of H2AX and CENP-F are related to Results and Methods and should not be in Introduction.

B) The detailed discussion of BER and NER is largely irrelevant to the paper.

C) Information largely missing from Introduction includes:
(i) LIG4 and XL4 human mutations
(ii) XRCC4 mouse phenotypes
(iii) XRCC4 and LIG4 structural and functional biology

D) Errors in the Introduction include:
(i) The authors probably do not mean to discuss or imply lesions or repair of ssDNA. Instead, the topics under consideration affect one of the two strands of a DNA duplex, not ssDNA.
(ii) "error-free" not "free-error"

6) Other experimental and methodological details:

A) It is never specified what "normal (a.k.a wt) fibroblasts" are, where they came from, or how comparable they might be to the patients cells. It is understood that it might not be possible have a truly matched cell pair, but it is important to evaluate (and highlights the potential value of a complementation experiment of patient cells with wt XRCC4).

B) Are these primary fibroblasts? Transformed? Early or late passage? I do not believe the handling or preparation of the patient fibroblasts is described beyond a statement of their culture medium.

7) Other text issues:

A) Avoid unnecessary abbreviations like WES and WB - spelling out whole-exome sequencing and Western blot is preferred in the one or two places the terms are used.

B) Case report II-2: Should be "He did not report".

C) Should probably be dbSNP, not dsSNPs.

D) "on cDNA extracted from ... fibroblasts" does not make sense. Cells have mRNA, not cDNA, so you cannot extract cDNA from fibroblasts.

E) "DNAse-treated cDNA samples" does not make sense. Treating cDNA with DNAse would obviously be a bad idea!

1st Revision - authors' response 28 February 2015

***** Reviewer's comments *****

Referee #1 (Comments on Novelty/Model System):

It would give additional info if the mutant XRCC4 protein was studied in vitro to determine whether there is residual activity.

Referee #1 (Remarks):

Bee et al. describe in two monozygotic twins born from consanguineous parents an late-onset (adult-onset) of progressive encephalocardiomyopathy. Using WES they identified a homozygous nonsense mutation in the XRCC4 gene. The authors claim that this is the first XRCC4 mutation described in human. However, earlier this year, Shaheen et al. published in Genome Research a patient with primordial dwarfism due to a mutation in XRCC4 (NM_003401.3: c.127T>C, p.Trp43Arg). This paper should be referred and discussed in the current manuscript. The clinical description is still highly relevant, but it is not the first XRCC4 deficient patient. This manuscript

gives a detailed study on the effects of the XRCC4 mutation on NHEJ and HR.

We thank the Reviewer for having drawn our attention to this work. Nevertheless, we note that Shaheen et al. considered *XRCC4* as a candidate gene for Primordial dwarfism but they did not perform any experiment to validate the deleterious role of the identified missense mutation (for instance using biological samples from patients). They just showed an obvious and largely expected finding, namely that *XRCC4* deficiency by RNAi results in impaired DNA damage repair.

In addition, the family reported by Shaheen et al is highly consanguineous and has a peculiar ethnic background, so it is not implausible that a subject can show a homozygous variant, extremely rare in public databases (4/120000 in Exac), but its pathogenicity has not been proven. Moreover, the segregation of the variant within the family is not reported.

We added a sentence in the discussion about the above reported paper, but we think that our variant is still the first unequivocally proven mutation in *XRCC4*.

1. In its current form the case report are rather extensive. The authors might consider to shorten them.

We shortened the case reports a bit but because the clinical findings associated with this *XRCC4* loss of function mutation are both novel and somehow unexpected, we do think important and interesting for the readership of EMBO Mol Med to report a detailed description of the neurological and extra-neurological features found in our patients.

2. Via RT-PCR and western blot analysis, a strongly reduced level of XRCC4 transcripts was detected, but protein could not be detected. Is this mutation regarded as a null mutant or can residual activity be expected? To address this point, the mutation needs to be cloned in an expression vector and activity needs to be measured. This would give additional info for correct interpretation of the mutation.

Previous and new WB experiments, incorporated in the present version of the paper, clearly demonstrate that both the full-length protein (as expected) and the predicted truncated R225* proteins are completely absent in patients' specimens. In this revised version we added WB experiments confirming that an anti-XRCC4 antibody able to detect the in-vitro synthesized truncated XRCC4 polypeptide, detects no immune-reactive band in mutant cells. As a consequence of this experimental evidence, we think that any information obtained from the overexpression of the truncated form (whether it is active or inactive) is neither necessary nor useful to explain the observed phenotype in cells where the truncated form is simply absent.

3. The authors did not find any immunological abnormalities in both patients, although immunodeficiency was expected based on the function of XRCC4. Could you speculate on how this could be explained e.g. redundancy or other mechanisms in the context of V(D)J recombination?

In the previous version of the paper we already hypothesized that compensatory mechanisms could explain the absence of immunological abnormalities ("*Taken together, our findings suggest that some vicarious mechanisms, only partly relying on the activation of the A-NHEJ pathway, makes the XRCC4 protein remarkably dispensable in humans, at least to warrant immunological proficiency and anti-cancer surveillance*").

In the revised version we added a WB experiment showing that, despite the lack of XRCC4, LIG4 is reduced but not absent in patients' cells. These findings can explain the milder phenotype observed in our mutant cells and, possibly, the mild clinical presentation of our patients. Our results are different with previous data obtained in *XRCC4* KO fibroblasts, the creation of which, however, is not reported (Ghezraoui et al. 2014).

4. Have patients with similar clinical characteristics been tested for mutations in XRCC4? The paper would be strengthened if more patients could be included.

In our cohort of patients, we did not find any individual with the same clinical presentation. We also performed exome sequencing analysis in tens of patients with various neurological impairments or multisystem disorders (encephalo and cardiomyopathies) but we have never found any deleterious variant in *XRCC4*.

Minor points:
1. The readability of Figure 5 can be improved if the percentages are also given in the figure.

We changed Figure 5 with Table 1 (reporting all the percentages) because we think it is more informative.

Referee #2 (Comments on Novelty/Model System):

Zeviani and coworkers use throughout the manuscript cells derived from two patients to draw most of their conclusions. The authors should complement these cells derived from the patients with the cDNA coding for XRCC4 to confirm that the observed phenotype is a direct consequence of the nonsense mutation found on XRCC4 in the two patients reported. Indeed they also report in the patients several additional homozygous mutations potentially deleterious that are not tested in the manuscript.

Referee #2 (Remarks):

Zeviani and co-workers report here the identification of the first human syndrome caused by a mutation in the XRCC4 gene creating a premature stop codon. The main known function for the XRCC4 protein is to stabilize DNA ligase IV, a protein mediating the final ligation step of the main DNA repair pathway of DNA double-strand breaks (DSB): Non Homologous End Joining (NHEJ). Several other human syndromes have already been reported to be caused by mutations in proteins of the NHEJ repair machinery such as Artemis and XLF/Cernnunos. Inactivating mutations in NHEJ proteins usually result in radiosensitivity, cancer predisposition and severe combined immunodeficiency (SCID) due to the essential role of NHEJ in the processes of V(D)J recombination and class-switch. Strikingly, the two patients reported here do not display immunodeficiency nor radiosensitivity nor cancer predispositions but instead a progressive encephalocardiomyopathy. When Zeviani and co. performed cellular analysis of fibroblasts derived from these patients they observed only a moderate repair defects, in contrast to what have been reported for XLF deficient cells for example (Buck et al. Cell 2006; Ahnesorg et al. Cell 2006). The most striking observation was a change in the balance between the different DNA double-strand break repair pathways with a higher proportion of DNA double-strand breaks being repaired by homologous recombination and by an alternative, slower and PARP-dependant DSB repair pathway known as alt-NHEJ or b-NHEJ. While the findings presented here are novel and provocative, they are also in direct contradiction with a recent work describing a complete knock-out of XRCC4 in human cells (Ghezraoui et al. 2014 Mol Cell). Indeed a clear NHEJ-dependant DNA repair defect is observed in these cells (similar to DNA Ligase IV knock out). In addition, other approaches (siRNA or shRNA) have also previously shown that reducing the level of XRCC4 in human cells leads to a repair defect that correlates with the efficiency of the siRNA/shRNA. In fact, siRNA against XRCC4 are frequently used as a positive control in DNA repair assays (see for example Ahnesorg et al. Cell 2006; Meerang et al. Nat Cell Biol 2011).
Therefore, from the data presented, two hypotheses have to be tested to explain this discrepancy (the absence of a clear DNA repair defect):
- the mutation reported might not be fully inactivating but instead hypomorphic.
- or one of the other homozygous and potentially inactivating mutations identified might compensate for the inactivation of XRCC4
Provided that the experiments necessary to test both hypotheses are conclusively performed (see below), the manuscript would be suitable for publication in EMBO Molecular Medicine. The quality of some experiments should also be improved (cell cycle distribution analysis) to reach the standards of EMBO Mol Med.

Major comments:

- Despite the level of XRCC4 being strongly reduced as shown in Fig1, a truncated version of XRCC4 might still be expressed and the reported phenotypes would therefore result from the hypomorphic nature of the mutation. Indeed it has been reported that a form of XRCC4 truncated for most of its c-terminus (XRCC4 1-250) is still able to complement the radiosensitivity of XRCC4 deficient cells (Koch et al. EMBO J 2004; should be cited in the manuscript); and XRCC4 1-225 still contains the DNA ligase IV interaction region (aa 173-195). To confirm that XRCC4 is not expressed on the form of a fragment, a new XRCC4 blot should be provided showing smaller proteins, together with the position of molecular weight markers.

We performed new WB analysis to demonstrate that the truncated form is absent using an antibody proven to detect the in-vitro synthesized truncated species.

- More importantly, since the main known function for XRCC4 is the stabilization of the DNA ligase IV protein, it is crucial to check the level of DNA ligase IV in patient cells as compared to control cells. If the levels are unaffected, or moderately affected, this would support the hypomorphic nature of the mutation and explain the lack of immunodeficiency in these patients, Different labs have developed anti-DNA Ligase IV antibodies that can be requested to perform this crucial control.

We used a commercially available antibody against LIG4 and observed only a partial reduction of LIG4 which, as suggested by the reviewer, can explain the “mild” clinical presentation of our patients (see also answer to rev#1).

- Since several additional homozygous and potentially inactivating mutations have also been discovered in these patients it is crucial to check that the main phenotypes reported here are due to the mutation on XRCC4, especially the reported imbalance between the DNA repair pathways. A rescue experiment in patient cells with the cDNA coding for human XRCC4 should be performed to check if the phenotypes observed in the patient cell lines (especially Rad51 foci and impact of the PARP inhibitor) are rescued by the re-expression of XRCC4. This is a standard experiment when attributing a phenotype to a mutation.

Unfortunately the cells from patients grow very slowly and do show signs of senescence; they cannot be used for any type of rescue experiment (transfection, viral transduction). However, the main message and novelty of this report is the fact that, in spite of the complete absence of XRCC4, cells maintain a residual competence to repair DNA double strand breaks that is compatible, in humans, with life. Therefore, we think that rescue experiments based on the re-expression of the wt gene address a secondary point in respect to the conceptual novelty of the work.

- knock-down experiments (siRNA/shRNA) should be performed on the patient cells to analyse if a further reduction of XRCC4 level (even if undetectable) would lead to an additional radiosensitivity. It has to be noted that knocking-down XRCC4 in human cells using siRNA have been used as a positive control in several works. In Ahnesorg et al. Cell 2006 for example, siRNA XRCC4 gives a sensitivity to ionizing radition similar to siRNA against XLF.

We have demonstrated by RT-PCR that patients’ fibroblasts have a reduction of *XRCC4* transcript (<<10%) similar or higher than any siRNA experiments; moreover, the remaining transcripts carry the nonsense mutation, hence it cannot lead to any protein production.

For these reasons, we think it is implausible that the suggested experiment could give us any further information.

- Zeviani and co. also identified at least two other proteins carrying potentially inactivating mutations (RSL1D1 and RDH8) and at least one of them has a link with the DNA damage response (RSL1D1, that "acts as a pro-apoptotic regulator in response to DNA damage" (Uniprot)). These mutations might compensate for the loss of XRCC4. If the authors failed to show that the mutation reported in XRCC4 is hypomorphic (see above), they should try to complement the patient cells with the cDNA of RSL1D1 and RDH8 to test if a reexpression of the wild-type form of these proteins radiosensitise the mutant cells but not the wild type cells.

As reported above, complementation experiments are not possible in our mutant cells, due to their poor growth.

Minor comments:

- most experiments do not include a positive control, such as the use of another human cell line deficient for another NHEJ component. This would greatly help to appreciate the sensitivity of the assays used here (maybe the mild effect of the mutation on DNA repair is just related to a technical problem) and provide a point of comparison. XLF deficient cell lines have been described (Ahnesorg P et al. Cell 2006 and Buck et al. Cell 2006).

We do not have any positive control but the experiments performed to show defective DNA repair are well established, published in numerous papers and mastered by the people in the laboratory of Prof. Celotti in Padova.

- the cell cycle analysis in Fig. 5 should be repeated with a more accurate quantification of S-phase index based on a specific staining of replicating cells (BrdU or EdU staining).

To have an accurate quantification of cells in S phase it would be better, as suggested by the Referee, to identify replicating cells by BrdU or EdU staining. However, the aim of the experiment reported in Table 1 was to evaluate the amount of cycling cells, in which the rejoining of DNA DSBs can be correctly performed by the HR system. Since 92-94% of XRCC4m/m cells were in G1 phase (as determined in three independent experiments analyzing 25.000 cells/each), we considered not very important to have a precise S-phase index. In any case, we modify the description of data reported in Table 1, underlining the size of G1 cell fraction and the missed activation of G2 block in the irradiated XRCC4m/m cells.

In our experimental conditions (Table 1) the major part of the cells was in G1 phase, as detected by analyzing DNA content. Before irradiation, 88.62% ± 1.86% of $XRCC4^{wt}$ fibroblasts was in G1 phase and 6.41% ± 0.35% in G2/M-phases; 24h after irradiation, G2 cells increased up to 11.09% ± 0.80%, probably because of G2-checkpoint activation. In $XRCC4^{m/m}$ non-irradiated cells the percentages of G1 phase was 92.23% ± 0.43% and that of G2/M phases 5.25% ± 1.47%. At 24 h after irradiation, $XRCC4^{m/m}$ cells in G1 phase were 93.91% ± 0.33% and those in G2/M were unchanged (5.44% ± 0.77%), probably because of failure of G2-checkpoint activation.

- sequence of primers used for RT-PCR should be provided

Added. We used two couple of primers; both located in the coding region upstream the nonsense mutation.

- to allow further studies to take place, authors should engage in establishing immortalized derivatives of the fibroblast cells used here.

As mentioned earlier, the conditions of patients' cells made it impossible their immortalization.

- p12: Zeviani and co. discuss about the "Cernnunos syndrome" and give its OMIM ID as 611290. Both information are incorrect: the syndrome is named the "NHEJ1 syndrome" and its OMIM ID is 611291. The ID 611290 refers to the XLF gene itself in the OMIM database.

We apologize for these inaccuracies that have now been amended.

- in few instances inappropriate publications are cited. For example:
+ Riballo and al. 2009 did not show that XRCC4 interaction with DNA ligase IV is required to stabilize DNA ligase IV. Instead, this was reported in Bryans et al. Mutat Res 1999 (CHO cells) and later confirmed in human in several report (e.g. Wu et al. J Biol Chem 2007).
+ Corneo et al. 2007 was not the first publication describing alternative NHEJ. This was reported in Wang et al. Nucleic Acids Research 2003 and other reports (Audebert et al. 2004).

We thank the reviewer for these clarifications. We changed the references accordingly.

Referee #3 (Comments on Novelty/Model System):

XRCC4-mutant patients are very novel, and cells from the patients are an appropriate model. Studies can be improved as discussed, and the medical impact is relevant to a small but growing subset of patients with DNA damage response defects.

Referee #3 (Remarks):

Bee et al. report intriguing findings in identical twins sharing a mild syndrome characterized by neurological, cardiac and developmental symptoms. Whole exome sequencing identified a clearly consequential homozygous mutation, consistent with consanguinity, leading to a premature stop codon in the XRCC4 gene involved in the nonhomologous end joining (NHEJ) DNA repair pathway. Remarkably, the patients did not show some expected phenotypes, in particular immunodeficiency, typically associated with NHEJ deficiency, and the cellular DSB repair defect was also relatively mild.

These findings are of potentially substantial interest to both human geneticists and DNA repair biologists. One the one hand, they might provide an explanation as to why XRCC4-deficient humans have not been identified among cohorts used to find patients with defects in the related LIG4 and XLF genes. However, it is those patients, and an extensive literature documenting the phenotypes of XRCC4-deficient cells, that make it challenging to understand the current results and patients. In general, it is plausible that the identified XRCC4 mutation is a major part of the cause of the patients' disease (although no other alleles are pursued). However, it is less certain that they have as complete of an XRCC4 deficiency as suggested. The truncation would leave a large portion of the XRCC4 protein intact, and in particular the portion that would likely support its continued dimerization and interactions with LIG4 and XLF. It would thus seem possible that this is a hypomorphic allele, in contrast to the complete loss of XRCC4 function suggested (even in the face of the potential for nonsense-mediated decay). In addition to some important text and other improvements, the paper would benefit from a more rigorous evaluation of the potential for residual XRCC4 expression and function.

Nonsense-mediated decay was confirmed by RT-PCR experiments in fibroblasts from both patients, carried out in two different laboratories. The levels of XRCC4 mRNA detected by this quantitative method is <10%. The mutation predicts a highly deleterious change in the protein (truncation at aminoacid position 225 (out of 336 residues composing the full-length protein); the complete absence of the mutant protein was confirmed by WB analysis using an antibody proven to detect the in vitro synthesized truncated species. Albeit the observed dysfunction is less severe than expected (at least compared to previous works on XRCC4 KO cellular/animal models), our experimental data on patients' cells clearly show the complete loss of XRCC4.

1) The critical issue is whether there really is a complete absence of XRCC4 protein. If the protein is truly absent, then the results would represent a substantial departure from previously described XRCC4 and NHEJ biology. This possibility is real and not discounted, and could reveal an interesting facet of human-specific NHEJ biology, but such a claim requires a rigorous demonstration beyond what is supplied. The results will be of interest even if the allele proves to be hypomorphic, but would be generally more consistent with expectations.

A) The Western blot should be repeated and presented:
(i) in a manner that allows the reader to examine both the position of the wild-type protein and the predicted position of the mutant protein, i.e. the whole blot should be shown, with molecular weight markers.
(ii) at a substantially greater "over-exposure" to show potentially low level proteins.
(iii) with at least one other, and ideally a polyclonal, antibody to corroborate results and guard against unanticipated false negative results.

(iv) ideally with an in vitro generated protein of the form anticipated and/or some other source of XRCC4 protein to allow determination of the blot sensitivity (how much protein could be detected?).

We performed WB experiments according to all the requests of rev#3. Two different polyclonal antibodies were tested, and in vitro generated polypeptides corresponding to the full length and the truncated forms were used as controls. The antibody from S. Cruz is against a peptide in the c-terminal region of XRCC4 and thus recognizes only the full-length protein; the antibody from Abcam was obtained using the full-length human XRCC4 as the antigen and recognizes both the full-length and the truncated XRCC4 polypeptides.

Both antibodies detected a band at the apparent MW of 55KDa in control cells that corresponds to the electrophoretic mobility of the synthesized full-length protein. Although the predicted MW of the protein is 38KDa, this experimental evidence is in agreement also with datasheets of diverse commercial antibodies, suggesting a peculiar structural conformation or a partial denaturation that alters the electrophoretic mobility of XRCC4.

B) There is a nearly complete absence of information presented as to the presumptive protein-level effects of any residual amount of mutant XRCC4 protein. This should be corrected, including the addition of a figure descriptive of the location of the mutation relative to the known protein domains. There is abundant information to consider that most notably includes crystallographic and functional biochemistry of wild-type and mutant proteins. This is not inconsequential, as there is an active recruitment of NHEJ repair proteins to DSBs, and even a minor "undetectable" amount of XRCC4 protein might be sufficient to supply a borderline NHEJ function consistent with the observations.

(Nearly) everything is possible in Nature but we would like to emphasize that the mRNA of mutant *XRCC4* was almost undetectable, and that the predicted truncated protein, which was clearly detected by a polyclonal antibody when synthesized in vitro, was completely absent even after prolonged exposure in all mutant samples we could analyze. We would like to maintain a position by which if experiments carried out according to the best laboratory practice do give certain results, the experimentalist scientists engaged in the project must discuss the results they obtained, rather than speculate that, in spite of the evidence obtained by their work, other interpretations are possible.

C) Although it moves beyond patient cells, it might be informative to establish a human XRCC4 knock-out/down cell system and ask whether the mutant allele could complement observed phenotypes.

The overexpression of the mutant allele in a KO cell model would not mirror what we observed in patients' cells, because in these cells the level of *XRCC4* mRNA is extremely low.

2) It is standard practice in DNA repair literature to show example photographic images of cell foci (H2AX and Rad51) to allow the reader to judge the quality and nature of results.

We added exemplifying images as requested.

3) The DNA content figure is not particularly interesting or informative.

Figure 5 was removed and substituted with a table.

4) The inclusion of the a-NHEJ-directed experiments is good and worthwhile, but at points results are over-stated with respect to the nature and precision of the manipulation and its effect on a-NHEJ. 3-AB is a reasonable experiment, but cannot be considered a highly targeted impairment of a-NHEJ.

Unfortunately the cells from patients grow slowly and are "suffering" and can't be used for any type of rescue experiment (transfection, viral transduction). Thus, to test the involvement of A-NHEJ in repairing DNA double strand breaks in patient fibroblasts we used a PARP-1 inhibitor. The results obtained from this experiment show, although indirectly, that this DNA repair system is used only in mutant fibroblasts. However, we agree with the Referee that the comment on these data has been over-stated. Accordingly, we removed panel C from Figure 6 and modified the last paragraph of the Results.

From the number of γ-H2AX foci in presence or absence of PARP-1 inhibitor (Fig. 6), we can roughly estimate the percentage of DSBs induced by γ-ray irradiation that were rejoined by the A-NHEJ. Albeit indirectly, our data suggested that the involvement of the A-NHEJ factors in repairing DSBs was irrelevant in $XRCC4^{wt}$ cells (5% ± 3% of DSBs repaired within 24 h), while it was clearly significant in $XRCC4^{m/m}$ cells (19% ± 4%).

5) Unlike most of the paper, the Introduction is poorly presented. It includes a large amount of irrelevant or misplaced information and lacks many topics of relevance to understanding the Results that follow. There are additionally some errors.

A) The detailed discussion of H2AX and CENP-F are related to Results and Methods and should not be in Introduction.

We moved this info in the results section.

B) The detailed discussion of BER and NER is largely irrelevant to the paper.

We removed this part.

C) Information largely missing from Introduction includes:
(i) LIG4 and XL4 human mutations
(ii) XRCC4 mouse phenotypes
(iii) XRCC4 and LIG4 structural and functional biology

D) Errors in the Introduction include:
(i) The authors probably do not mean to discuss or imply lesions or repair of ssDNA. Instead, the topics under consideration affect one of the two strands of a DNA duplex, not ssDNA.
(ii) "error-free" not "free-error"

Corrected

6) Other experimental and methodological details:

A) It is never specified what "normal (a.k.a wt) fibroblasts" are, where they came from, or how comparable they might be to the patients cells. It is understood that it might not be possible have a truly matched cell pair, but it is important to evaluate (and highlights the potential value of a complementation experiment of patient cells with wt XRCC4).

Wt fibroblasts were from healthy adult subjects, obtained from our biobank.

B) Are these primary fibroblasts? Transformed? Early or late passage? I do not believe the handling or preparation of the patient fibroblasts is described beyond a statement of their culture medium.

We used primary skin fibroblasts at passages 3-7 (either from patients or controls). Standard protocols were used for preparation of fibroblasts from skin biopsies (e.g. Establishment of Fibroblast Cultures, Current Protocols in Cell Biology (1998), by Akira Takashima).

7) Other text issues:

A) Avoid unnecessary abbreviations like WES and WB - spelling out whole-exome sequencing and Western blot is preferred in the one or two places the terms are used.

B) Case report II-2: Should be "He did not report".

C) Should probably be dbSNP, not dsSNPs.

D) "on cDNA extracted from ... fibroblasts" does not make sense. Cells have mRNA, not cDNA, so you cannot extract cDNA from fibroblasts.

E) "DNAse-treated cDNA samples" does not make sense. Treating cDNA with DNAse would obviously be a bad idea!

Changed, thank you.

Additional author correspondence 13 March 2015

We think we have to draw your attention to a paper just published this month March 2015 in the American Journal of Human Genetics entitled "Mutations in the NHEJ Component XRCC4 Cause Primordial Dwarfism" by Murray et al.

Some findings presented in this paper clearly overlap with those reported in the paper "Genetic ablation of human XRCC4 is associated with adult-onset progressive encephalocardiomyopathy" that we have re-submitted to EMBO Mol Med (revised version uploaded last 28th of February).

However, there are several data in either work, which are original, so as we think these two papers can nicely complement each other. For instance, despite the presence at birth of cryptorchidism, hypotelorism, short limbs, and short stature, our patients were not classified as having primordial dwarfism and the main clinical feature was an adult-onset progressive encephalocardiomyopathy; the detailed description of their adult phenotype could represent the expected clinical outcome also for the patients reported by Murray et al., that are all still children.

Interestingly, the data reported by the AJHG Authors allow us to answer to some Reviewers' requests that at the moment of resubmission we could not answer: for instance, the identification of additional patients; and to some hypotheses formulated by the reviewers, for instance that a second mutation in our twins could counteract the "lethal" effect of the XRCC4 absence) made by reviewers we were not able to reply.

The main message from both papers is that, in contrast with expectations based on previous findings from Xrcc4 KO mouse, which is embryo-lethal, in humans nonsense mutations in XRCC4 are compatible with life and XRCC4 protein is remarkably dispensable. Notably, in our paper we obtained some evidence on the possible molecular mechanisms that are activated in the absence of XRCC4.

Based on these considerations, we think that our paper nicely complements the one just published, and hope it will be accepted soon by EMBO Mol Med as a further contribution to understand the function of XRCC4 in humans.

2nd Editorial Decision 14 March 2015

Thank you for the submission of your revised manuscript to EMBO Molecular Medicine. We have now received the enclosed reports from the referees that were asked to re-assess it.

You will see that referee 2 saw the recent paper you mentioned, along with a second one, which overall make the total number of patients with XRCC4 mutations up to 9, with almost no immunodeficiency reported. We agree with you that these papers support your findings and made us realise that indeed, some of the still requested experiments by the referees (rescue experiment for ex.) appear not absolutely necessary. Nevertheless, we would like to ask you to revise your article in the following way:

-Incorporate all 3 papers mentioned by the referees (1 and 2) in your article and discuss them accordingly.
-The expression level of DNA ligase IV remain an issue (referees 2 and 3). Please discuss appropriately why this might be; especially in light of other XRCC4 mutant patients who express reduced ligase IV levels as expected from the literature. Please do not forget to amend your material and methods to indicate which antibody anti-Lig4 you used and at which dilution.
-As suggested by referee 2, you have to rewrite in appropriate sections to reflect that the 2 patients you analysed are not the 1st ones with a proven XRCC4 mutation anymore.
-Tune down the conclusions regarding the "absence" of XRCC4 mutant protein as we agree with the referees that a Western Blot cannot be sufficient for such a claim, and a hypomorphic mutant is not excluded by the presented experiments.
-Please add a figure panel to exactly map the mutation as seen in the Am J Hum Genet paper (referee 3).

Please reply in a point-by-point rebuttal letter to all issues raised by the referees.

Please make sure to address all editorial points listed below in order to accelerate the process should your paper be accepted as it is my understanding, that we should move fast for your paper to remain timely as referee 1 said.

I look forward to seeing a revised form of your manuscript as soon as possible (ideally, within 2 weeks).

***** Reviewer's comments *****

Referee #1 (Comments on Novelty/Model System):

The XRCC4 deficiency is still highly novel and more reports appear in literature or are presented during scientific meetings. It is really striking that an XRCC4 deficiency does not result in immunodeficiency like other NHEJ defects and this is also well discussed by Bee et al. Both the clinical and biological description of the patients will be a valuable contribution to literature to get a good impression of the apparent clinical heterogeneity of NHEJ defects.
The paper is timely, also because of the recent identified new NHEJ factor PAXX (a paralog of XRCC4 and XLF) which is was recently published in Science.

Referee #1 (Remarks):

I regret that the authors decided not to perform in vitro cloning of the mutant and in vitro testing of the protein. The authors are right that in vivo the protein cannot be detected by Western blot. However, it is not excluded that a small trace of mutant protein (that cannot be detected by Western blot with the given sensitivity) still has some residual activity. If there is no residual activity at all, one would expect that is a null mutation. Based on the embryonal lethality of XRCC4 knock out mice, I would expect that this mutation is hypomorphic.

The other points were well addressed.

Referee #2 (Comments on Novelty/Model System):

In my previous review, to improve the adequacy of the model system, I requested from Zeviani and co. to re-express Xrcc4 cDNA in the patient cells to confirm that some of molecular phenotypes they describe are related to XRCC4 defect itself and not to the other homozygous mutations they also identify in these patients. Zeviani and co. rejected most of the experiments requested since they claim it is not possible to immortalize their cells. In the DNA repair field, different labs have been successful in immortalizing cell lines from patients with even more severe defects. For example, the lab from de Villartay immortalised cells out of XLF/Cernunnos-deficient patients (XLF is another NHEJ factor) that presented microcephaly and immnodeficiency (Buck et al. Cell 2006). In that

case, de Villartay and co. used SV40 and telomerase immortalization a method that they also used previoulsy to immortalize Artemis-deficient cells (Artemis is another NHEJ factor). In the same study, they successfully complemented these cells with XLF cDNA, which was an essential part of their work that confirmed that the cellular defects observed where caused by XLF deficiency. Since then these cell lines have been of great help to the DNA repair community and were used to study the functions and regulations of XLF. I suggest that Zeviani and co. use this study as a framework to immortalize their own patient cells and perform a rescue experiment.

Referee #2 (Remarks):

Major comments:
- The Novelty of the findings reported by Zeviani and co. has decreased since two publications recently came out and reported the identification of several new patients that have mutations in XRCC4 gene leading to different human syndromes associated with growth and developmental defects without immunodeficiency (de Bruin et al. J Clin Endocrinol Metab 2015 PMID: 25742519; Murray JE et al. Am J Hum Genet 2015 PMID: 25728776). Therefore the novelty claimed in Zeviani's manuscript has to be tuned down and these two publications cited and discussed in appropriate sections of the manuscript. For example, the sentence "These are the first patients reported with experimentally proven XRCC4 mutations." has to be removed from the abstract and this kind of statement " we identified the first, ever reported human XRCC4 nonsense mutation " from the main text.

- In Murray JE et al. Am J Hum Genet 2015 several new patients carrying homozygous mutation in the XRCC4 gene are now identified. Some of these mutations lead to a reduction into XRCC4 protein level. In these cells, despite the XRCC4 level being still higher than in the cells reported by Zeviani, the DNA Ligase IV is strongly reduced in contrast to what reports Zeviani using a commercial anti-DNA Ligase IV antibody. I suspect that the antibody used by Zeviani and co. does not recognize DNA Ligase IV itself but another protein running at a similar size. In the DNA repair field, it is well known that most commercial anti-DNA ligase IV antibodies are unspecific, it is why I suggested in my previous comments to Zeviani and co. to contact groups that have been successful in generating specific anti-Lig4 antibody. It is crucial that Zeviani and co. repeat the analysis of DNA Ligase IV levels in their cell lines using a specific antibody and including in this new experiment a specificity control for this new antibody for example on the form of extracts from DNA Ligase IV deficient cells (HCT116 Lig4-/- and N114 Lig4-/- have been described and can be requested from the corresponding labs). I have to emphasize that this is a critical point: in all previous publication regarding DNA Ligase IV, it has been observed that a decrease in XRCC4 level leads to a decrease in DNA Ligase IV level.

- a rescue experiment has to performed by re-expressing Xrcc4 in the patient cells. In an elegant study, the lab from de Villartay immortalized cells out of XLF/Cernunnos-deficient patients that present microcephaly and immnodeficiency (Buck et al. Cell 2006). The method used is SV40 and telomerase immortalisation. In the same study, they successfully complemented these immortalized cells with XLF cDNA, an essential part of their work confirming that the cellular defects observed were caused by XLF deficiency. Since then these cell lines have been of great help to the DNA repair community and were successfully used to study the function and regulation of XLF. Zeviani and co. should use this study as a framework to immortalize their own patient cells and perform a rescue experiment. The impact of XRCC4 reexpression in patient cells should be analysed at the level of gamma-H2AX and RAD51 foci formation and resolution.

Minor comments:
- the newly identified NHEJ factor PAXX should be added to the introduction section (Oshi et al. Science 2015).

Referee #3 (Comments on Novelty/Model System):

XRCC4-mutant patients are novel, and cells from the patients are an appropriate model. The medical impact is relevant to a small but growing subset of patients with DNA damage response defects.

Referee #3 (Remarks):

Bee et al. present an improved version of their manuscript describing human patients with a nonsense mutation of the NHEJ gene XRCC4. Changes are in assembly of the manuscript and marked improvements in the Western blot analyses. However, additional experimental systems examining the function of the mutated protein are not provided.

The central question is whether orthogonal approaches are necessary or helpful in the face of apparent lack of expression of the truncated protein form. To be sure, the improved Western blots, taken in conjunction with mRNA analysis, support the idea that very little mutant XRCC4 protein is present in cultured patient fibroblasts. Strikingly, LIG4 protein is present at nearly normal levels, different than observed in previous cell systems lacking XRCC4. There is clearly merit to the argument that the patient fibroblast experiments are therefore informative as to the consequence of the mutations and in documenting the existence of previously unanticipated relationships between XRCC4, LIG4 and NHEJ. The findings will be of interest as presented.

On the other hand, there remain reasons that further experimentation would inform the consequences of the truncation/stop codon, in these patients and in general. First, expression and repair results are only presented for fibroblasts, while interpretation of phenotypes such as immunodeficiency depends on other cell types that may not behave the same and may utilize the truncated protein more effectively. Second, the authors did not appreciate the force of prior comments; it is not a distant theoretical possibility that NHEJ proteins can preferentially accumulate at DSBs in a functional manner even when very poorly expressed, it has been observed (e.g http://www.sciencedirect.com/science/article/pii/S156878641400247X). There is an important potential difference between "not present" and "not detectable by Western blot", especially when even the longest exposure of the Western blots show only modest bands for full-length XRCC4 from patient cells. Finally, independent of interpreting these patients, further experimentation is simply worthwhile as it could tell us more about the function of truncated XRCC4 proteins in potentially a variety of cell types. I am not overly concerned about "over expression" because certain result patterns might still be highly informative. Moreover, with current technologies it is conceivable that one might make knock-in mutations.

I finally repeat that the paper is still deficient in describing the precise nature and location of the protein truncation with respect to XRCC4 structural biology. The most precise comment made is "loss of one third of the protein at the C-terminus". This isn't sufficient and should be described more clearly and assisted by a figure. As above, the authors rely on the idea that it doesn't matter since the protein is absent, but again the latter has only been documented in one cell type. Readers involved in studying DSB repair will expect to find more substantive information as to the exact nature of the mutation.

2nd Revision - authors' response 20 March 2015

***** Reviewer's comments *****

Referee #1 (Comments on Novelty/Model System):

The XRCC4 deficiency is still highly novel and more reports appear in literature or are presented during scientific meetings. It is really striking that an XRCC4 deficiency does not result in immunodeficiency like other NHEJ defects and this is also well discussed by Bee et al. Both the clinical and biological description of the patients will be a valuable contribution to literature to get a good impression of the apparent clinical heterogeneity of NHEJ defects.
The paper is timely, also because of the recent identified new NHEJ factor PAXX (a paralog of XRCC4 and XLF) which is was recently published in Science.

Referee #1 (Remarks):

I regret that the authors decided not to perform in vitro cloning of the mutant and in vitro testing of the protein. The authors are right that in vivo the protein cannot be detected by Western blot. However, it is not excluded that a small trace of mutant protein (that cannot be detected by Western blot with the given sensitivity) still has some residual activity. If there is no residual activity at all, one would expect that is a null mutation. Based on the embryonal lethality of XRCC4 knock out mice, I would expect that this mutation is hypomorphic.

It has been already demonstrated that truncated version of XRCC4 may still maintain residual activity and can interact with LIG4 (Koch et al. 2004 EMBO J). However, we think that information on the "quality/functionality" of the mutant protein cannot be separated from the "quantity" in order to have a physiological, and not just theoretical, explanation.
We changed the statement "absence of XRCC4 protein" with "undetectable level of XRCC4".
In the discussion we added a paragraph on this issue:
"Although the persistence of small amounts of mutant protein retaining some residual activity cannot be excluded, neither we nor others were able to detect any trace of truncated XRCC4 species, at least in patients' fibroblasts. This does not exclude the possibility that, in other cell types, mRNA decay or stability of the truncated protein may be different and these cells may utilize the truncated protein more effectively."

The other points were well addressed.

Referee #2 (Comments on Novelty/Model System):

In my previous review, to improve the adequacy of the model system, I requested from Zeviani and co. to re-express Xrcc4 cDNA in the patient cells to confirm that some of molecular phenotypes they describe are related to XRCC4 defect itself and not to the other homozygous mutations they also identify in these patients. Zeviani and co. rejected most of the experiments requested since they claim it is not possible to immortalize their cells. In the DNA repair field, different labs have been successful in immortalizing cell lines from patients with even more severe defects. For example, the lab from de Villartay immortalised cells out of XLF/Cernunnos-deficient patients (XLF is another NHEJ factor) that presented microcephaly and immnodeficiency (Buck et al. Cell 2006). In that case, de Villartay and co. used SV40 and telomerase immortalization a method that they also used previoulsy to immortalize Artemis-deficient cells (Artemis is another NHEJ factor). In the same study, they successfully complemented these cells with XLF cDNA, which was an essential part of their work that confirmed that the cellular defects observed where caused by XLF deficiency. Since then these cell lines have been of great help to the DNA repair community and were used to study the functions and regulations of XLF. I suggest that Zeviani and co. use this study as a framework to immortalize their own patient cells and perform a rescue experiment.

We thank the reviewer for his/her suggestion; however, according to the editorial strategy to accelerate the process (2 weeks), there is no time to obtain immortalized fibroblasts and then to perform complementation experiments. Moreover, the reports on additional patients with XRCC4 mutations (most of which with nonsense or frame-shift mutations) make the rescue experiments not necessary.

Referee #2 (Remarks):

Major comments:
- The Novelty of the findings reported by Zeviani and co. has decreased since two publications recently came out and reported the identification of several new patients that have mutations in XRCC4 gene leading to different human syndromes associated with growth and developmental defects without immunodeficiency (de Bruin et al. J Clin Endocrinol Metab 2015 PMID: 25742519; Murray JE et al. Am J Hum Genet 2015 PMID: 25728776). Therefore the novelty claimed in Zeviani's manuscript has to be tuned down and these two publications cited and discussed in appropriate sections of the manuscript. For example, the sentence "These are the first patients reported with experimentally proven XRCC4 mutations." has to be removed from the abstract and

this kind of statement " we identified the first, ever reported human XRCC4 nonsense mutation " from the main text.

We would like to draw the attention of this Reviewer to the fact that our paper was submitted months ago, and we were completely unaware of concurrent work on the same topic by colleagues with whom we had no contact whatsoever. Therefore, we consider our own contribution as absolutely original. Of course, in the revised version the two papers are mentioned and the statements on the “first patients” were removed; in the discussion we compared our results with those from these two recent papers.

- In Murray JE et al. Am J Hum Genet 2015 several new patients carrying homozygous mutation in the XRCC4 gene are now identified. Some of these mutations lead to a reduction into XRCC4 protein level. In these cells, despite the XRCC4 level being still higher than in the cells reported by Zeviani, the DNA Ligase IV is strongly reduced in contrast to what reports Zeviani using a commercial anti-DNA Ligase IV antibody. I suspect that the antibody used by Zeviani and co. does not recognize DNA Ligase IV itself but another protein running at a similar size. In the DNA repair field, it is well known that most commercial anti-DNA ligase IV antibodies are unspecific, it is why I suggested in my previous comments to Zeviani and co. to contact groups that have been successful in generating specific anti-Lig4 antibody. It is crucial that Zeviani and co. repeat the analysis of DNA Ligase IV levels in their cell lines using a specific antibody and including in this new experiment a specificity control for this new antibody for example on the form of extracts from DNA Ligase IV deficient cells (HCT116 Lig4-/- and N114 Lig4-/- have been described and can be requested from the corresponding labs). I have to emphasize that this is a critical point: in all previous publication regarding DNA Ligase IV, it has been observed that a decrease in XRCC4 level leads to a decrease in DNA Ligase IV level.

As stated above, according to the editorial strategy to accelerate the process (2 weeks), there is no time to perform these experiments.
Nevertheless, in the present version, we replaced the WB image of the LIG4 (which was possibly exposed too much) with a new image, obtained from the same filter at a shorter exposure. In this image (as well as in several other independent replicates) there is a clear reduction of LIG4 in patients’ fibroblasts (40% of control mean by densitometric analysis). We think it is unlikely that the signal, which appears consistently reduced in patients compared to controls, may be unspecific.
We have no definite explanation for this experimental finding but it can possibly explain the milder phenotype observed in our patients.

- a rescue experiment has to performed by re-expressing Xrcc4 in the patient cells. In an elegant study, the lab from de Villartay immortalized cells out of XLF/Cernunnos-deficient patients that present microcephaly and immnodeficiency (Buck et al. Cell 2006). The method used is SV40 and telomerase immortalisation. In the same study, they successfully complemented these immortalized cells with XLF cDNA, an essential part of their work confirming that the cellular defects observed were caused by XLF deficiency. Since then these cell lines have been of great help to the DNA repair community and were successfully used to study the function and regulation of XLF. Zeviani and co. should use this study as a framework to immortalize their own patient cells and perform a rescue experiment. The impact of XRCC4 reexpression in patient cells should be analysed at the level of gamma-H2AX and RAD51 foci formation and resolution.

See the first answer to referee #2.

Minor comments:
- the newly identified NHEJ factor PAXX should be added to the introduction section (Oshi et al. Science 2015).

Done.

Referee #3 (Comments on Novelty/Model System):

XRCC4-mutant patients are novel, and cells from the patients are an appropriate model. The

medical impact is relevant to a small but growing subset of patients with DNA damage response defects.

Referee #3 (Remarks):

Bee et al. present an improved version of their manuscript describing human patients with a nonsense mutation of the NHEJ gene XRCC4. Changes are in assembly of the manuscript and marked improvements in the Western blot analyses. However, additional experimental systems examining the function of the mutated protein are not provided.

The central question is whether orthogonal approaches are necessary or helpful in the face of apparent lack of expression of the truncated protein form. To be sure, the improved Western blots, taken in conjunction with mRNA analysis, support the idea that very little mutant XRCC4 protein is present in cultured patient fibroblasts. Strikingly, LIG4 protein is present at nearly normal levels, different than observed in previous cell systems lacking XRCC4. There is clearly merit to the argument that the patient fibroblast experiments are therefore informative as to the consequence of the mutations and in documenting the existence of previously unanticipated relationships between XRCC4, LIG4 and NHEJ. The findings will be of interest as presented.

On the other hand, there remain reasons that further experimentation would inform the consequences of the truncation/stop codon, in these patients and in general. First, expression and repair results are only presented for fibroblasts, while interpretation of phenotypes such as immunodeficiency depends on other cell types that may not behave the same and may utilize the truncated protein more effectively. Second, the authors did not appreciate the force of prior comments; it is not a distant theoretical possibility that NHEJ proteins can preferentially accumulate at DSBs in a functional manner even when very poorly expressed, it has been observed (e.g http://www.sciencedirect.com/science/article/pii/S156878641400247X). There is an important potential difference between "not present" and "not detectable by Western blot", especially when even the longest exposure of the Western blots show only modest bands for full-length XRCC4 from patient cells. Finally, independent of interpreting these patients, further experimentation is simply worthwhile as it could tell us more about the function of truncated XRCC4 proteins in potentially a variety of cell types. I am not overly concerned about "over expression" because certain result patterns might still be highly informative. Moreover, with current technologies it is conceivable that one might make knock-in mutations.

See all the previous answers.

I finally repeat that the paper is still deficient in describing the precise nature and location of the protein truncation with respect to XRCC4 structural biology. The most precise comment made is "loss of one third of the protein at the C-terminus". This isn't sufficient and should be described more clearly and assisted by a figure. As above, the authors rely on the idea that it doesn't matter since the protein is absent, but again the latter has only been documented in one cell type. Readers involved in studying DSB repair will expect to find more substantive information as to the exact nature of the mutation.

According to reviewer's suggestion, we added a figure with a schematic view of the protein (Fig. 1B) and a paragraph with some info on the functional domains.

…with loss of one third of the protein at the C-terminus. This portion of the protein contains a low-complexity domain with several sites that can be phosphorylated by DNA-PK, causing loss of DNA end-bridging (Mahaney et al, 2013), whereas the head domain (aa 1-115) forming the hydrophobic core, and the stalk (aa 115-203), a coiled-coil domain important for dimerization and interaction with LIG4, are upstream the truncating mutation (Fig 1B). A form of XRCC4 truncated for most of its c-terminus (aa 1-250) was reported to be able to complement the radiosensitivity of XRCC4 deficient cells (Koch et al, 2004).

3rd Editorial Decision 23 March 2015

Thank you for resubmitting your revised manuscript to EMBO Molecular Medicine. I am pleased to inform you that we will be able to accept your manuscript pending the following final amendment:

Main text

Please double check in the discussion section (newly added text). I could not find the reference Shaheen et al 2014 in the reference list. Could you please double check? Also you mention 3 papers published during revision/reviewing of your article. Murray et al, De Bruin et al, but Shaheen et al was published in February last year. Besides, while I agree that this paper should be mentioned as 1 patient is referenced, no clinical data are available, only the genomic sequence if I am not mistaken. Therefore, I don't think it provides the same insights as the other 2. Please revise accordingly.

I look forward to reading a new revised version of your manuscript as soon as possible.
